# Geranylated Coumarins From Thai Medicinal Plant *Mammea siamensis* With Testosterone 5α-Reductase Inhibitory Activity

**DOI:** 10.3389/fchem.2020.00199

**Published:** 2020-03-20

**Authors:** Toshio Morikawa, Fenglin Luo, Yoshiaki Manse, Hidemi Sugita, Shunsuke Saeki, Saowanee Chaipech, Yutana Pongpiriyadacha, Osamu Muraoka, Kiyofumi Ninomiya

**Affiliations:** ^1^Pharmaceutical Research and Technology Institute, Kindai University, Osaka, Japan; ^2^Faculty of Agro-Industry, Rajamangala University of Technology Srivijaya, Nakhon Si Thammarat, Thailand; ^3^Faculty of Science and Technology, Rajamangala University of Technology Srivijaya, Nakhon Si Thammarat, Thailand

**Keywords:** *Mammea siamensis*, mammeasin, 5α-reductase inhibitor, geranylated coumarin, calophyllaceae

## Abstract

Geranylated coumarin constituents, kayeassamin I (**1**) and mammeasins E (**2**) and F (**3**) were newly isolated from the methanol extract of the flowers of *Mammea siamensis* (Calophyllaceae) originating in Thailand, along with five known isolates, such as mammea E/BC (**23**), deacetylmammea E/AA cyclo D (**31**), deacetylmammea E/BB cyclo D (**32**), mammea A/AA cyclo F (**34**), and mammea A/AC cyclo F (**35**). These compounds (**1**–**3**) were obtained as an inseparable mixture (*ca*. 1:1 ratio) of the 3″*R* and 3″*S* forms, respectively. Among the isolated coumarins from the extract, mammeasins E (**2**, 22.6 μM), A (**4**, 19.0 μM), and B (**5**, 24.0 μM), kayeassamins E (**9**, 33.8 μM), F (**10**, 15.9 μM), and G (**11**, 17.7 μM), surangin C (**13**, 5.9 μM), and mammeas A/AA (**17**, 19.5 μM), E/BB (**22**, 16.8 μM), and A/AA cyclo F (**34**, 23.6 μM), were found to inhibit testosterone 5α-reductase.

## Introduction

The Calophyllaceae plant *Mammea siamensis* (Miq.) T. Anders. is a small evergreen tree distributed in Thailand (locally called “Sarapi” or “Saraphi”), Laos, Cambodia, Vietnam, and Myanmar. The flowers of this plant have traditionally been used as a heart tonic, fever-lowering, and enhancement of appetite in Thailand (Morikawa et al., 2012; Tung et al., 2013; Ninomiya et al., 2016; Sangkaruk et al., 2017). Previous chemical studies on the flowers (Kaweetripob et al., 2000; Prachyawarakorn et al., 2000, 2006a; Mahidol et al., 2002; Morikawa et al., 2012; Ninomiya et al., 2016), seeds (Laphookhieo et al., 2006, 2007), twigs (Poobrasert et al., 1998; Prachyawarakorn et al., 2006a,b), and bark (Ngo et al., 2010) of *M. siamensis* reported on the isolation of several coumarins and xanthones, etc. With regard to the biological studies on *M. siamensis* and its constituents, cytotoxicity, antiproliferative, and apoptotic effects against several tumor and cancer cell lines (Ngo et al., 2010; Tung et al., 2013; Noysang et al., 2014; Uto et al., 2016; Sangkaruk et al., 2017), suppressive effects on inducible nitric oxide synthase expression in RAW264.7 cells (Morikawa et al., 2012), and aromatase inhibitory activity (Ninomiya et al., 2016; Tanabe et al., 2017) have been reported. Further separation of the constituents in the extract resulted in the isolation of three geranylated coumarins, kayeassamin I (**1**) and mammeasins E (**2**) and F (**3**). Here, we conducted the isolation and structural verification of **1**–**3**, as well as examined the testosterone 5α-reductase inhibitory activity of its coumarin constituents (**1**–**35**), including five new isolates, such as mammea E/BC (**23**), deacetylmammea E/AA cyclo D (**31**), deacetylmammea E/BB cyclo D (**32**), mammea A/AA cyclo F (**34**), and mammea A/AC cyclo F (**35**).

## Materials and Methods

### General Experimental Procedures

The following instruments were used to obtain physical data: a SEPA-300 digital polarimeter (Horiba Ltd., Kyoto, Japan, *l* = 5 cm) for specific rotations; an UV-1600 spectrometer (Shimadzu Co., Kyoto, Japan) to record UV spectra; a FTIR-8100 spectrometer (Shimadzu Co.) to measure IR spectra; a JNM-ECA800 (800 MHz), JNM-ECA700 (700 MHz), JNM-ECA500 (500 MHz), and JNM-ECS400 and JNM-AL400 (400 MHz) spectrometers (JEOL Ltd., Tokyo, Japan) to determine ^1^H NMR spectra; JNM-ECA800 (200 MHz), JNM-ECA700 (175 MHz), JNM-ECA500 (125 MHz), and JNM-ECS400 and JNM-AL-400 (100 MHz) spectrometers (JEOL Ltd.) to record ^13^C NMR spectra in CDCl_3_ at room temperature (25°C) with tetramethylsilane as an internal standard; an Exactive Plus Orbitrap mass spectrometer (Thermo Fisher Scientific Inc., Waltham, MA, USA) to measure ESIMS and HRESIMS; an HPLC detector, SPD-10A*vp* UV-Vis (Shimadzu Co.); and Cosmosil 5C_18_-MS-II (Nacalai Tesque, Inc., Kyoto, Japan) HPLC columns (4.6 mm i.d. × 250 mm and 20 mm i.d. × 250 mm) for analytical and preparative purposes, respectively.

The following materials and experimental conditions were used for the column chromatography (CC): normal-phase silica gel CC, silica gel 60N (Kanto Chemical Co., Ltd., Tokyo, Japan; 63–210 mesh, spherical, neutral); reversed-phase ODS CC, Chromatorex ODS DM1020T (Fuji Silysia Chemical, Ltd., Aichi, Japan; 100–200 mesh); TLC, pre-coated TLC plates with silica gel 60F_254_ (Merck, Darmstadt, Germany, 0.25 mm, normal-phase) and silica gel RP-18 WF_254S_ (Merck, Darmstadt, Germany, 0.25 mm, reversed-phase); reversed-phase HPTLC, pre-coated TLC plates with silica gel RP-18 WF_254S_ (Merck, 0.25 mm); detection was performed by spraying 1% Ce(SO_4_)_2_-10% aqueous H_2_SO_4_, followed by heating.

### Plant Material

The flowers of *Mammea siamensis* were collected from the Nakhonsithammarat Province, Thailand, in September 2006, as described previously (Morikawa et al., 2012; Ninomiya et al., 2016). The plant material was identified by one of the authors (Y. P.). A voucher specimen (2006.09. Raj-04) for this plant has been deposited in our laboratory.

### Extraction and Isolation

Dried flowers of *M. siamensis* (1.8 kg) were extracted three times with MeOH under reflux for 3 h. Evaporation of the combined extracts under reduced pressure afforded the MeOH extract (463.7 g, 25.66%). An aliquot (413.7 g) of the extract was partitioned into an EtOAc–H_2_O (1:1, v/v) mixture to furnish an EtOAc-soluble fraction (110.34 g, 6.84%) and an aqueous phase. An aliquot (89.45 g) of the EtOAc-soluble fraction was subjected to normal-phase silica gel CC [3.0 kg, *n*-hexane–EtOAc (10:1 → 7:1 → 5:1, v/v) → EtOAc → MeOH] to give 11 fractions [Fr. 1 (3.05 g), Fr. 2 (2.86 g), Fr. 3 (11.71 g), Fr. 4 (1.62 g), Fr. 5 (4.15 g), Fr. 6 (6.29 g), Fr. 7 (2.21 g), Fr. 8 (2.94 g), Fr. 9 (10.23 g), Fr. 10 (11.17 g), and Fr. 11 (21.35 g)]. Fraction 5 (4.15 g) was subjected to reversed-phase silica gel CC [120 g, MeOH–H_2_O (80:20 → 85:15, v/v) → MeOH → acetone] to afford six fractions [Fr. 5-1 (115.7 mg), Fr. 5-2 (2789.8 mg), Fr. 5-3 (515.4 mg), Fr. 5-4 (430.0 mg), Fr. 5-5 (119.2 mg), and Fr. 5-6 (110.0 mg)] as reported previously (Ninomiya et al., 2016). Fraction 5-2 (517.0 mg) was purified by HPLC [Cosmosil 5C_18_-MS-II, MeOH−1% aqueous AcOH (85:15, v/v)] to give mammea A/AC cyclo F (**35**, 4.6 mg, 0.0019%) (Morel et al., 1999; Prachyawarakorn et al., 2000; Guilet et al., 2001) together with mammeas A/AA (**17**, 101.2 mg, 0.0418%), A/AC (**19**, 112.9 mg, 0.0466%), A/AA cyclo D (**24**, 2.7 mg, 0.0011%), E/BC cyclo D (**29**, 14.0 mg, 0.0058%), and E/BD cyclo D (**30**, 1.8 mg, 0.0015%) (Mahidol et al., 2002). Fraction 5-3 (515.4 mg) was purified by HPLC [Cosmosil 5C_18_-MS-II, MeOH−1% aqueous AcOH (85:15, v/v)] to give mammea A/AA cyclo F (**34**, 13.2 mg, 0.0010%) (Prachyawarakorn et al., 2000; Guilet et al., 2001) together with **19** (45.6 mg, 0.0035%), **24** (14.9 mg, 0.0011%), mammeas A/AB cyclo D (**25**, 46.4 mg, 0.0035%) and A/AC cyclo D (**26**, 30.1 mg, 0.0023%). Fraction 6 (6.29 g) was subjected to reversed-phase silica gel CC [200 g, MeOH–H_2_O (80:20 → 90:10 → 95:5, v/v) → MeOH → acetone] to afford 10 fractions [Fr. 6-1 (44.7 mg), Fr. 6-2 (157.2 mg), Fr. 6-3 (928.8 mg), Fr. 6-4 (3117.0 mg), Fr. 6-5 (128.8 mg), Fr. 6-6 (487.1 mg), Fr. 6-7 (230.8 mg), Fr. 6-8 (280.5 mg), Fr. 6-9 (102.9 mg), and Fr. 6-10 (96.5 mg)] as reported previously (Morikawa et al., 2012; Ninomiya et al., 2016). Fraction 6-4 (536.2 mg) was purified by HPLC [Cosmosil 5C_18_-MS-II, MeOH−1% aqueous AcOH (90:10, v/v)] to give kayeassamin I (**1**, 7.2 mg, 0.0032%) (Win et al., 2008b), mammeasin E (**2**, 16.5 mg, 0.0073%), and **35** (11.0 mg, 0.0049%) together with mammeasins A (**4**, 65.8 mg, 0.0293%) and B (**5**, 21.6 mg, 0.0096%), surangin B (**12**, 58.2 mg, 0.0259%), **17** (17.0 mg, 0.0076%), mammea A/AB (**18**, 10.7 mg, 0.0048%), and **19** (112.6 mg, 0.0501%). Fraction 7 (2.21 g) was subjected to reversed-phase ODS CC [47.0 g, MeOH-H_2_O (60:40 → 80:20 → 90:10, v/v) → MeOH → acetone] to afford five fractions [Fr. 7-1 (187.3 mg), Fr. 7-2 (912.0 mg), Fr. 7-3 (275.2 mg), Fr. 7-4 (30.0 mg), and Fr. 7-5 (44.0 mg)]. Fraction 7-2 (912.0 mg) was purified by HPLC [column: Cosmosil 5C_18_-MS-II, detection: UV (230 nm), mobile phase: MeOH-1% aqueous H_2_O (85:15, v/v)] to give mammeasin E/BC (**23**, 99.0 mg, 0.0076%) (Yang et al., 2005). Fraction 7-3 (275.2 mg) was purified by HPLC [Cosmosil 5C_18_-MS-II, UV (230 nm), MeOH-1% aqueous AcOH 85:15, v/v] to give **1** (52.1 mg, 0.0040%), **2** (34.1 mg, 0.0026%), and mammeasin F (**3**, 19.5 mg, 0.0015%). Fraction 9 (10.23 g) was subjected to reversed-phase silica gel CC [300 g, MeOH–H_2_O (80:20 → 90:10, v/v) → MeOH → acetone] to afford five fractions [Fr. 9-1 (2809.0 mg), Fr. 9-2 (5678.0 mg), Fr. 9-3 (385.9 mg), Fr. 9-4 (422.0 mg), and Fr. 9-5 (51.9 mg)] as reported previously (Morikawa et al., 2012; Ninomiya et al., 2016). Fraction 9-1 (544.5 mg) was purified by HPLC [Cosmosil 5C_18_-MS-II, MeOH−1% aqueous AcOH (85:15, v/v)] to give deacetylmammeas E/AA cyclo D (**31**, 1.3 mg, 0.0005%) (Mahidol et al., 2007) and E/BB cyclo D (**32**, 6.1 mg, 0.0023%) (Mahidol et al., 2007) together with kayeassamins E (**9**, 28.6 mg, 0.0113%), F (**10**, 98.7 mg, 0.0390%), and G (**11**, 43.4 mg, 0.0171%), deacetylmammea E/BC cyclo D (**33**, 18.6 mg, 0.0073%), and benzoic acid (10.9 mg, 0.0043%).

#### Kayeassamin I (1)

Pale yellow oil; ${\left[\alpha \right]}_{\text{D}}^{25}$ −50.4 (*c* 0.63, CHCl_3_) ${\left[\alpha \right]}_{\text{D}}^{23}$ −35.52 (*c* 0.90, CHCl_3_) (Win et al., 2008a)}; ^1^H and ^13^C NMR spectroscopic data (see Table 1); Negative-ion ESIMS *m/z* 439 [M – H]^−^; HRESIMS *m/z* 439.2116 (calcd for C_26_H_31_O_6_, 439.2115) (Figures S3–S7).

**Table 1:** ^1^H and ^13^C NMR spectroscopic data (CDCl_3_) for kayeassamin I (**1**).

**Position**	**1a^a^**		**1b^a^**		**1^b^**	
	**δ_**H**_**	**δ_**C**_**	**δ_**H**_**	**δ_**C**_**	**δ_**H**_**	**δ_**C**_**
2		159.6		159.6		159.6
3	6.60 (br s)	107.0	6.61 (d, 0.9)	107.2	6.61 (s)	107.0
4		160.6		160.6		160.6
4a		101.0		101.1		101.0
5		155.9		155.9		155.9
6		105.8		106.0		105.8
7		162.9		162.9		162.9
8		104.5		104.6		104.5
8a		157.2		157.2		157.3
1′	5.43 (br t, *ca*. 8)	71.8	5.43 (br t, *ca*. 8)	71.7	5.43 (d, 8.1)	71.8
2′	1.51, 1.95 (both m)	30.7	1.53, 1.96 (both m)	30.5	1.50, 1.97 (both m)	30.7
3′	1.11 (3H, t, 7.4)	10.2	1.09 (3H, t. 7.4)	10.1	1.13 (3H, t, 7.4)	10.2
2″		83.0		83.1		83.0
3″	5.53 (d, 10.2)	125.0	5.55 (d, 10.2)	124.8	5.54 (d, 10.0)	124.9
4″	6.78 (d, 10.2)	116.5	6.79 (d, 10.2)	116.6	6.79 (d, 10.0)	116.5
5″	1.71, 1.91 (both m)	41.6	1.71, 1.91 (both m)	41.9	1.90 (2H, m)	41.6
6″	2.09 (2H, m)	23.0	2.09 (2H, m)	23.2	2.09 (2H, m)	23.0
7″	5.06 (qt, 0.9, 7.1)	123.1	5.06 (qt, 0.9, 7.1)	123.0	5.07 (t, 7.1)	123.1
8″		132.6		132.6		132.6
9″	1.64 (3H, d, 0.9)	25.6	1.67 (3H, d, 0.9)	25.6	1.64 (3H, s)	25.6
10″	1.55 (3H, s)	17.6	1.57 (3H, s)	17.7	1.55 (3H, s)	17.7
1^‴^		206.4		206.4		206.4
2^‴^	3.26 (2H, t, 7.1)	46.7	3.26 (2H, t, 7.1)	46.7	3.27 (2H, t, 7.1)	46.7
3^‴^	1.78 (2H, qt, 7.4, 7.1)	18.0	1.78 (2H, qt, 7.4, 7.1)	18.0	1.79 (2H, m)	18.1
4^‴^	1.04 (3H, t, 7.4)	13.8	1.03 (3H, t, 7.4)	13.8	1.05 (3H, m)	13.8
2″-CH_3_	1.52 (3H, s)	27.2	1.48 (3H, s)	27.5	1.51 (3H, s)	27.3
7–OH	14.47 (s)		14.47 (s)		14.48 (brs)	

a *Measured by 800 MHz for ^1^H NMR and 200 MHz for ^13^C NMR*.

b *Reported in Win et al. (2008b) by 400 MHz for ^1^H NMR and 100 MHz for ^13^C NMR*.

#### Mammeasin E (2)

Pale yellow oil; ${\left[\alpha \right]}_{\text{D}}^{25}$ −58.9 (*c* 0.12, CHCl_3_); UV (MeOH) λ_max_ nm (log ε): 223 (4.01), 278 (4.12), 302 (4.12); IR (KBr) ν_max_ cm^−1^: 1,740, 1,713, 1,613, 1,454, 1,408, 1,284, 1,126, 1,049; ^1^H and ^13^C NMR spectroscopic data (see Table 2); Negative-ion ESIMS *m/z* 453 [M – H]^−^; HRESIMS *m/z* 453.2272 (calcd for C_27_H_33_O_6_, 453.2272) (Figures S8–S12).

**Table 2:** ^1^H and ^13^C NMR spectroscopic data (CDCl_3_) for mammeasins E (**2**) and F (**3**).

**Position**	**2a^a^**		**2b^a^**		**3a^b^**		**3b^b^**	
	**δ_**H**_**	**δ_**C**_**	**δ_**H**_**	**δ_**C**_**	**δ_**H**_**	**δ_**C**_**	**δ_**H**_**	**δ_**C**_**
2		159.6		159.6		159.5		159.5
3	6.61 (d, 0.9)	107.0	6.59 (d, 1.0)	107.1	6.62 (d, 1.0)	107.1	6.61 (d, 1.0)	107.2
4		160.7		160.7		160.6		1560.5
4a		101.0		101.1		101.0		101.2
5		156.0		156.0		155.8		155.8
6		105.8		106.0		105.9		106.1
7		163.0		163.0		163.1		163.1
8		104.5		104.6		104.3		104.3
8a		157.1		157.1		157.0		156.9
1′	5.40 (br t, *ca*. 8)	71.8	5.40 (br t, *ca*.8)	71.7	5.43 (m)	71.8	5.43 (m)	71.8
2′	1.53, 1.96 (both m)	30.7	1.53, 1.96 (both m)	30.6	1.52, 1.96 (both m)	30.7	1.52, 1.96 (both m)	30.6
3′	1.12 (3H, t, 7.3)	10.2	1.09 (3H, t, 7.1)	10.1	1.12 (3H, t, 7.1)	10.2	1.10 (3H, t, 7.1)	10.1
2″		83.0		83.1		83.0		83.1
3″	5.53 (d, 10.2)	125.0	5.54 (d, 10.2)	124.9	5.54 (d, 10.2)	124.9	5.54 (d, 10.2)	124.8
4″	6.79 (d, 10.2)	116.5	6.78 (d, 10.2)	116.5	6.79 (d, 10.2)	116.6	6.79 (d, 10.2)	116.6
5″	1.71, 1.90 (both m)	41.6	1.71, 1.90 (both m)	41.8	1.71, 1.91 (both m)	41.7	1.71, 1.91 (both m)	41.9
6″	2.08 (2H, m)	23.0	2.08 (2H, m)	23.2	2.09 (2H, m)	23.0	2.09 (2H, m)	23.3
7″	5.06 (qt, 1.0, 7.1)	123.1	5.06 (qt, 1.0, 7.1)	123.0	5.06 (dt, 1.3, 7.1)	123.1	5.06 (qt, 1.3, 7.1)	123.0
8″		132.6		132.5		132.6		132.6
9″	1.64 (3H, d, 1.0)	25.5	1.67 (3H, d, 1.0)	25.6	1.64 (3H, br s)	25.6	1.67 (3H, br s)	25.6
10″	1.52 (3H, s)	17.6	1.54 (3H, s)	17.7	1.57 (3H, s)	17.6	1.57 (3H, s)	17.7
1^‴^		206.2		206.2		210.7		210.7
2^‴^	3.14 (2H, d, 6.7)	53.6	3.14 (2H, d, 6.7)	53.6	3.89 (m)	47.0	3.89 (m)	47.0
3^‴^	2.27 (m)	25.6	2.27 (m)	25.5	1.25 (3H, d, 6.7)	16.6	1.26 (3H, d, 6.7)	16.6
4^‴^	1.03 (3H, d, 6.6)	22.6	1.03 (3H, d, 6.6)	22.6	1.46, 1.89 (both m)	27.2	1.46, 1.89 (each m)	27.2
5^‴^	1.03 (3H, d, 6.6)	22.6	1.03 (3H, d, 6.6)	22.6	0.98 (3H, t, 7.5)	11.7	0.98 (3H, t, 7.5)	11.7
2^‴^-CH_3_	1.51 (3H, s)	27.3	1.48 (3H, s)	27.5	1.52 (3H, br s)	27.3	1.47 (3H, br s)	27.5
7-OH	14.51 (s)		14.51 (s)		14.44 (s)		14.44 (s)	

a *Measured by 700 MHz for ^1^H NMR and 175 MHz for ^13^C NMR*.

b *Measured by 800 MHz for ^1^H NMR and 200 MHz for ^13^C NMR*.

#### Mammeasin F (3)

Pale yellow oil; ${\left[\alpha \right]}_{\text{D}}^{25}$ −42.1 (*c* 0.45, CHCl_3_); UV (MeOH) λ_max_ nm (log ε): 224 (3.89), 298 (3.82); IR (KBr) ν_max_ cm^−1^: 1,732, 1,713, 1,605, 1,454, 1,381, 1,261, 1,126, 1,049; ^1^H and ^13^C NMR spectroscopic data (see Table 2); Negative-ion ESIMS *m/z* 453 [M – H]^−^; HRESIMS *m/z* 453.2287 (calcd for C_27_H_33_O_6_, 453.2272) (Figures S13–S17).

### DDQ Oxidation of Kayeassamin A (8) and Surangins C (13) and D (14)

A solution of kayeassamin A (**8**, 9.0 mg) in dry-toluene (2.0 mL) was treated with 2,3-dichloro-5,6-dicyano-*p*-benzoquinone (DDQ, 10.0 mg) and the solution stirred at room temperature (25°C) for 4 h. The aqueous solution was saturated with sodium hydrogen carbonate (NaHCO_3_) and extracted with EtOAc. The EtOAc extract was washed with brine then dried over anhydrous magnesium sulfate (MgSO_4_) and filtered. Removal of the solvent under reduced pressure gave a residue, which was purified by HPLC [Cosmosil 5C_18_-MS-II, MeOH−1% aqueous AcOH (85:15, v/v)] to give kayeassamin I (**1**, 3.8 mg, 46%). Through the similar procedure, mammeasin E (**2**, 3.3 mg, 38%) and mammeasin F (**3**, 2.0 mg, 17%) were obtained from surangins D (**14**, 9.6 mg) and C (**13**, 12.7 mg), respectively.

### Assay for Testosterone 5α-Reductase Inhibitory Activity

The experiment was performed in accordance with previously reported methods (Matsuda et al., 2001; Lee et al., 2012; Koseki et al., 2015) with slight modifications. In brief, the assay was performed in 48-well microplates (Sumitomo Bakelite Co., Ltd., Tokyo, Japan). The reaction solution was pre-incubated with or without a test sample (5 μL/well, dissolved in DMSO), in a potassium phosphate buffer (40 mM, pH 6.5, 490 μL/well) containing substrate (0.35 nmol of testosterone, Tokyo Chemical Industry Co., Ltd., Tokyo, Japan) and NADPH (10 nmol, Oriental Yeast Co., Ltd., Tokyo, Japan) at room temperature (25°C) for 20 min. The enzymatic reaction was initiated by the addition of rat liver S9 fractions (10 μL/well, dissolved in the phosphate buffer, 20.6 μg/well, Oriental Yeast Co., Ltd., Tokyo, Japan, lot no. 109031513) at 37°C for 30 min. After incubation, the reaction mixture was immediately heated in boiling water for 2 min to stop the reaction. Then the reaction solution of each well was transferred to a microtube and extracted with 500 μL of EtOAc. After the microtube was centrifuged (10,000 rpm, 5 min), an aliquot of each EtOAc phase (300 μL) was transferred into another tube. The solvent in the tube was evaporated and the residue was dissolved in 30 μL of acetonitrile containing an internal standard (I.S.) fludrocortisone acetate (20 μg/mL, Sigma-Aldrich, Co., LLC, St. Louis, USA). An aliquot of 2 μL was injected into the HPLC under the following conditions [Instrument: a series LC-20A Prominence HPLC system (Shimadzu Co., Kyoto, Japan); Detection: UV (254 nm); Column: Cosmosil 5C_18_-MS-II (Nakalai Tesque Inc., Kyoto, Japan, 5 μm particle size, 2.0 mm i.d. × 150 mm); Column temperature: 40°C; Mobile phase: MeOH–H_2_O (60:40, v/v); Flow rate: 0.2 mL/min; retention time: 13.5 min for testosterone and 8.0 min for I.S. A similar procedure that described above was carried out for the control tubes. The 5α-reductase inhibitory activity was determined from the following equation using the peak area ratios (*r* = testosterone/I.S.). Experiments were performed in triplicate or quadruple, and IC_50_ values were determined graphically. The 5α-reductase inhibitor finasteride (Tokyo Chemical Industry Co., Ltd., Tokyo, Japan) was used as a reference compound.

$$\begin{array}{l} \text{Inhibition}\left(\%\right)=\left[r\left(\text{T}\right)-r\left(\text{C}\right)/r\left(\text{B}\right)-r\left(\text{C}\right)\right]\times \;100 \end{array}$$

Control (C): enzyme (+), test sample (–); Test (T): enzyme (+), test sample (+); Blank (B): enzyme (–), test sample (+).

### Statistics

Values are expressed as mean ± S.E.M. One-way analysis of variance (ANOVA), followed by Dunnett's test, was used for statistical analysis. Probability (*p*) values < 0.05 were considered significant.

## Results and Discussion

### Effects of the Methanol Extract From the Flowers of *M. siamensis* on Testosterone 5α-Reductase

The male sex hormones, androgens, play a crucial role in the development, growth and function of the prostate, and other androgen-sensitive peripheral tissues. In the prostate gland, androgens are involved in benign prostatic hyperplasia and prostate cancer, as well as in skin disorders, such as acne, seborrhea, androgenic alopecia, and hirsutism. Among the androgens, testosterone is the most abundant in serum and secreted primarily by the testicles and ovaries. The enzyme steroid 5α-reductase catalyzes the conversion of testosterone to the most potent natural androgen, 5α-dihydrotestosterone (Yamana et al., 2010; Yao et al., 2011; Azzouni et al., 2012). Therefore, inhibition of testosterone 5α-reductase could be useful for the treatment of the above diseases. To date, three types of 5α-reductases, chronologically named types 1, 2, and 3 5α-reductases, have been described (Yamana et al., 2010; Azzouni et al., 2012; Titus et al., 2014). A type 2 and 3 5α-reductase inhibitor, finasteride, is currently marketed worldwide as a drug for benign prostatic hyperplasia and is also used in the treatment of hair loss (Heinzl, 1999; Tosti and Piraccini, 2000) and in the prevention of prostate cancer (Coltman et al., 1999). Therefore, 5α-reductase is considered a useful therapeutic target in the treatment and prevention of the above deceases. In particular, many heterocyclic compounds based on oxygen and nitrogen atoms often have good antiproliferative activity against a variety of solid tumor cell lines and are expected to be seeds of new anticancer agents (Sharma et al., 2018; Petel et al., 2019).

During our characterization studies on bioactive constituents from Thai natural medicines (Manse et al., 2017; Morikawa et al., 2018; Tanabe et al., 2018; Kobayashi et al., 2019), a methanol extract of the flowers of *M. siamensis* was found to inhibit 5α-reductase activity (IC_50_ = 2.4 μg/mL). In order to investigate new 5α-reductase inhibitors, we conducted a search for the bioactive constituents from the flowers of *M. siamensis*.

### Isolation

**Figure 1:**
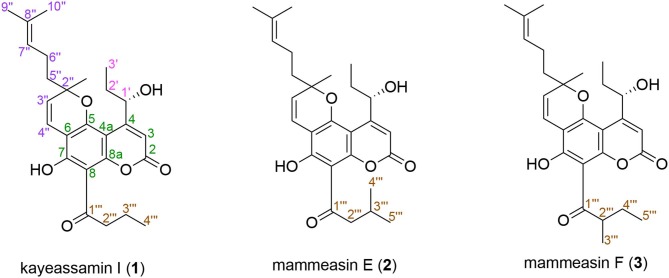
Structures of kayeassamin I **(1)** and mammeasins E **(2)** and F **(3)**.

**Figure 2:**
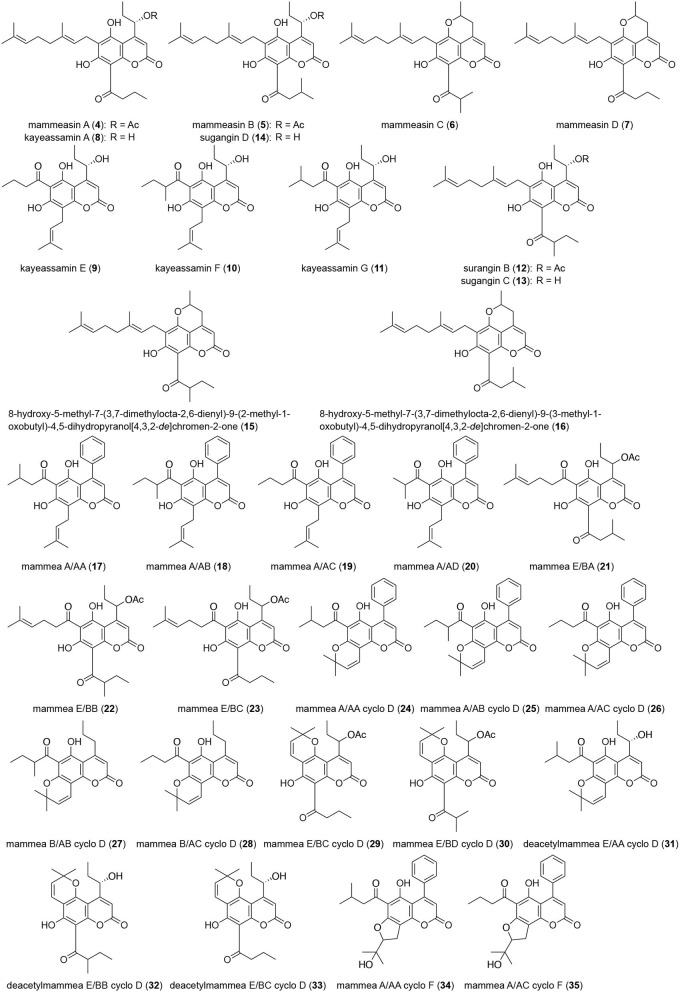
Coumarin constituents **(4–35)** from the flowers of *M. siamensis*.

In our previous report we described the isolation of 26 coumarins: mammeasins A (**4**, 0.0293%), B (**5**, 0.0115%), C (**6**, 0.0008%), and D (**7**, 0.0047%), kayeassamins A (**8**, 0.0578%), E (**9**, 0.0113%), F (**10**, 0.0390%), and G (**11**, 0.0171%), surangins B (**12**, 0.0271%), C (**13**, 0.0571%), and D (**14**, 0.0632%), 8-hydroxy-5-methyl-7-(3,7-dimethyl-octa-2,6-dienyl)-9-(2-methyl-1-oxobutyl)-4,5-dihydropyrano[4,3,2-*de*]chromen-2-one (**15**, 0.0015%), 8-hydroxy-5-methyl-7-(3,7-dimethyl-octa-2,6-dienyl)-9-(3-methyl-1-oxobutyl)-4,5-dihydropyrano[4,3,2-*de*]chromen-2-one (**16**, 0.0012%), mammeas A/AA (**17**, 0.0494%), A/AB (**18**, 0.0048%), A/AC (**19**, 0.1056%), A/AD (**20**, 0.0022%), E/BA (**21**, 0.0045%), E/BB (**22**, 0.0194%), A/AA cyclo D (**24**, 0.0035%), A/AB cyclo D (**25**, 0.0097%), A/AC cyclo D (**26**, 0.0109%), B/AB cyclo D (**27**, 0.0016%), B/AC cyclo D (**28**, 0.0062%), E/BC cyclo D (**29**, 0.0058%), and deacetylmammea E/BC cyclo D (**33**, 0.0073%), as described previously (Morikawa et al., 2012; Ninomiya et al., 2016). In the present study, we additionally isolated kayeassamin I (**1**, 0.0072%) and mammeasins E (**2**, 0.0099%) and F (**3**, 0.0015%), from the methanol extract of *M. siamensis* flowers as shown in Figure 1, together with six coumarins: mammeas E/BC (**23**, 0.0076%) and E/BD cyclo D (**30**, 0.0015%), deacetylmammeas E/AA cyclo D (**31**, 0.0005%) and E/BB cyclo D (**32**, 0.0023%), and mammeas A/AA cyclo F (**34**, 0.0010%) and A/AC cyclo F (**35**, 0.0068%), using normal-phase silica gel and reversed-phase ODS column chromatographic purification steps, and finally by HPLC (Figure 2).

### Structures of Kayeassamin I (1) and Mammeasins E (2) and F (3)

**Figure 3:**
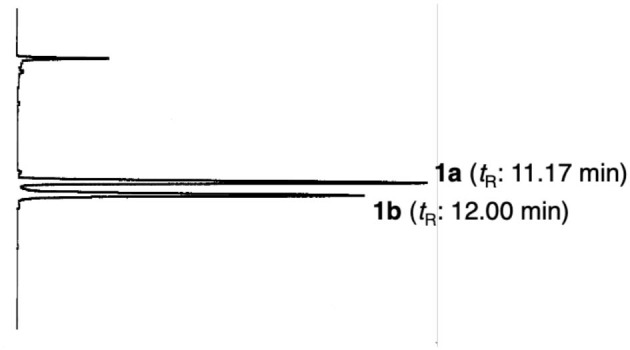
HPLC chromatogram of kayeassamin I **(1a, 1b)**. HPLC condition: column, Cosmosil 5C_18_-MS-II (250 × 4.6 mm, i.d.); detection, UV (254 nm); mobile phase, CH_3_CN−1% aqueous AcOH (90:10, v/v); flow rate, 1.0 mL/min; column temperature, r.t. (25°C).

Compound **1** was obtained as pale yellow oil with a negative optical rotation (${\left[\alpha \right]}_{\text{D}}^{25}$ −50.4 in CHCl_3_), and its molecular formula was deduced to be C_26_H_32_O_6_ by high-resolution ESIMS (HRESIMS) measurement. As shown in Figure 3, the HPLC analysis suggested that **1** was obtained as an inseparable mixture (*ca*. 1:1 ratio). The ^1^H and ^13^C NMR spectra spectroscopic properties (Table 1, CDCl_3_) of **1**, which were assigned with the aid of DEPT, DQF-COSY, HSQC, and HMBC experiments, were in accordance with those of kayeassamin I except for the observation of duplicate signals (**1a** and **1b**) measured by high resolution 800 MHz NMR spectrometer: two primary, a tertiary, and two vinyl methyls [**1a**: δ 1.04 (3H, t, *J* = 7.4 Hz, H_3_-4^‴^), 1.11 (3H, t, *J* = 7.4 Hz, H_3_-3′), 1.52 (3H, s, 2″-CH_3_), 1.55 (3H, s, H_3_-10″), 1.64 (3H, d, *J* = 0.9 Hz, H_3_-9″); **1b**: δ 1.03 (3H, t, *J* = 7.4 Hz, H_3_-4^‴^), 1.09 (3H, t, *J* = 7.4 Hz, H_3_-3′), 1.48 (3H, s, 2″-CH_3_), 1.57 (3H, s, H_3_-10″), 1.67 (3H, d, *J* = 0.9 Hz, H_3_-9″)], five methylenes [**1a**: δ 1.51, 1.95 (1H each, both m, H_2_-2′), 1.71, 1.91 (1H each, both m, H_2_-5″), 1.78 (2H, qt, *J* = 7.4, 7.1 Hz, H_2_-3^‴^), 2.09 (2H, m, H_2_-6″), 3.26 (2H, t, *J* = 7.1 Hz, H_2_-2^‴^); **1b**: δ 1.53, 1.96 (1H each, both m, H_2_-2′), 1.71, 1.91 (1H each, both m, H_2_-5″), 1.78 (2H, qt, *J* = 7.4, 7.1 Hz, H_2_-3^‴^), 2.09 (2H, m, H_2_-6″), 3.26 (2H, t, *J* = 7.1 Hz, H_2_-2^‴^)], a methine bearing an oxygen function [**1a**: δ 5.43 (1H, br t, *J* = *ca*. 8 Hz, H-1′); **1b**: δ 5.43 (1H, br t, *J* = *ca*. 8 Hz, H-1′)], four olefinic protons [**1a**: δ 5.06 (1H, qt, *J* = 0.9, 7.1 Hz, H-7″), 5.53 (1H, d, *J* = 10.2 Hz, H-3″), 6.60 (1H, br s, H-3), 6.78 (1H, d, *J* = 10.2 Hz, H-4″); **1b**: δ 5.06 (1H, qt, *J* = 0.9, 7.1 Hz, H-7″), 5.55 (1H, d, *J* = 10.2 Hz, H-3″), 6.61 (1H, d, *J* = 0.9 Hz, H-3), 6.79 (1H, d, *J* = 10.2 Hz, H-4″)], and a hydrogen-bonded hydroxy proton [**1a**: δ 14.47 (1H, s, 7-OH); **1b**: δ 14.47 (1H, s, 7-OH)]. This evidence allowed us to revise the structure of kayeassamin I as a mixture (**1a** and **1b**) of *ca*. 1:1 inseparable stereoisomers in the 2″ position. The absolute configuration of the 1′-position in **1** has been assumed to be *S* by comparison of the optical rotation with that of similar compounds (Win et al., 2008b). To confirm the stereochemistry, we carried out chemical correlation between **1** and kayeassamin A (**8**), which has been reported to be in the 1′*S* form by the modified Mosher's method (Win et al., 2008a). Thus, oxidation of **8** with 2,3-dichloro-5,6-dicyano-*p*-benzoquinone (DDQ) gave **1**. Consequently, the absolute configuration in the 1′ position of **1** was confirmed to be *S*.

Mammeasin E (**2**) was also obtained as an inseparable mixture (*ca*. 1:1 ratio, Figure S1) with a negative optical rotation (${\left[\alpha \right]}_{\text{D}}^{25}$ −58.9 in CHCl_3_). In the negative-ion ESIMS of **2**, a quasimolecular ion peak was observed at *m/z* 453 [M – H]^−^, and HRESIMS analysis indicated the molecular formula was C_27_H_34_O_6_. The ^1^H and ^13^C NMR spectra (Table 2, CDCl_3_) of **2** were similar to those of **1**, except for the signals due to the 3-methyl-1-oxobutyl moiety in the 8-position [**2a**: δ 1.03 (6H, d, *J* = 6.6 Hz, H_3_-4^‴^ and H_3_-5^‴^), 2.27 (1H, m, H-3^‴^), 3.14 (2H, d, *J* = 6.7 Hz, H_2_-2^‴^); **2b**: δ 1.03 (6H, d, *J* = 6.6 Hz, H_3_-4^‴^ and H_3_-5^‴^), 2.27 (1H, m, H-3^‴^), 3.14 (2H, d, *J* = 6.7 Hz, H_2_-2^‴^)] instead of the 1-oxobutyl moiety of **1**. As shown in Figure S2, the connectivity of the quaternary carbons in **2** were elucidated on the basis of DQF-COSY and HMBC experiments. Thus, the DQF-COSY experiment on **2** indicated the presence of the following partial structures: C-1′-C-3′; C-3″-C-4″; C-5″-C-7″; and C-2^‴^–C-5^‴^ shown in bold lines. In the HMBC experiment, long-range correlations were observed between the following proton and carbon pairs: H-3 [**2a**: δ 6.61 (1H, d, *J* = 0.9 Hz); **2b**: δ 6.59 (1H, d, *J* = 1.0 Hz)] and C-2 (**2a**: δ_C_ 159.6; **2b**: δ_C_ 159.6), C-4a (**2a**: δ_C_ 101.0; **2b**: δ_C_ 101.1); the hydrogen-bonded hydroxy proton [**2a**: δ 14.51 (1H, s); **2b**: δ 14.51 (1H, s)] and C-6 (**2a**: δ_C_ 105.8; **2b**: δ_C_ 106.0), C-7 (**2a**: δ_C_ 163.0; **2b**: δ_C_ 163.0), C-8 (**2a**: δ_C_ 104.5; **2b**: δ_C_ 104.6); H-1′ [**2a**: δ 5.40 (1H, br t, *J* = *ca*. 8 Hz); **2b**: δ 5.40 (1H, br t, *J* = *ca*. 8 Hz)] and C-3 (**2a**: δ_C_ 107.0; **2b**: δ_C_ 107.1), C-4a; H-3^‴^ [**2a**: δ 5.53 (1H, d, *J* = 10.2 Hz); **2b**: δ 5.54 (1H, d, *J* = 10.2 Hz)] and C-6, C-2″ (**2a**: δ_C_ 83.0; **2b**: δ_C_ 83.1), 2″-*C*H_3_ (**2a**: δ_C_ 27.3; **2b**: δ_C_ 27.5); H-4″ [**2a**: δ 6.79 (1H, d, *J* = 10.2 Hz); **2b**: δ 6.78 (1H, d, *J* = 10.2 Hz)] and C-5 (**2a**: δ_C_ 156.0; **2b**: δ_C_ 156.0), C-6; H_2_-5″ [**2a**: δ 1.71, 1.90 (1H each, both m); **2b**: δ 1.71, 1.90 (1H each, both m)] and C-2″, 2″-*C*H_3_; H-7″ [**2a**: δ 5.06 (1H, qt, *J* = 1.0, 7.1 Hz); **2b**: δ 5.06 (1H, qt, *J* = 1.0, 7.1 Hz)] and C-9″ (**2a**: δ_C_ 25.5; **2b**: δ_C_ 25.6), C-10″ (**2a**: δ_C_ 17.6; **2b**: δ_C_ 17.7); H-9″ [**2a**: δ 1.64 (3H, d, *J* = 1.0 Hz); **2b**: δ 1.67 (3H, d, *J* = 1.0 Hz)] and C-7″ (**2a**: δ_C_ 123.1; **2b**: δ_C_ 123.0), C-8″ (**2a**: δ_C_ 132.6; **2b**: δ_C_ 132.5), C-10″; H-10″ [**2a**: δ 1.52 (3H, s); **2b**: δ 1.54 (3H, s)] and C-7″-9″; and H_2_-2^‴^ and C-1^‴^ (**2a**: δ_C_ 206.2; **2b**: δ_C_ 206.2). On the other hand, the molecular formula of mammeasin F (**3**) was determined to be the same as that of **2**, C_27_H_34_O_6_, by HRESIMS measurement. The ^1^H and ^13^C NMR spectroscopic properties (Table 2, CDCl_3_) of **3**, which were observed to be duplicate signals caused by its inseparable mixture (*ca*. 1: 1 ratio, Figure S1), were quite similar to those of **2** except for the signals due to the 2-methyl-1-oxobutyl moiety in the 8-position [**3a**: δ 0.98 (3H, t, *J* = 7.5 Hz, H_3_-5^‴^), 1.25 (3H, d, *J* = 6.7 Hz, H_3_-3^‴^), 1.46, 1.89 (each 1H, both m, H_2_-4^‴^), 3.89 (1H, m, H_2_-2^‴^); **3b**: δ 0.98 (3H, t, *J* = 7.5 Hz, H_3_-5^‴^), 1.26 (3H, d, *J* = 6.7 Hz, H_3_-3^‴^), 1.46, 1.89 (each 1H, both m, H_2_-4^‴^), 3.89 (1H, m, H_2_-2^‴^)]. Finally, **2** and **3** were derived by DDQ oxidation of surangins D (**14**) (Ngo et al., 2010) and C (**13**) (Verotta et al., 2004; Yagi et al., 2006), respectively. Based on this evidence, the stereostructures of **2** and **3** were determined to be as shown.

### Effects of Coumarin Constituents of the Flowers of *M. siamensis* on Testosterone 5α-Reductase

To characterize the active constituents of this plant material, the inhibitory effects of 30 isolates (**1**–**13**, **17**–**20**, **22**–**29**, **31**–**35**) against 5α-reductase were examined. As shown in Table 3, mammeasins E (**2**, 22.6 μM), A (**4**, 19.0 μM), and B (**5**, 24.0 μM), kayeassamins E (**9**, 33.8 μM), F (**10**, 15.9 μM), and G (**11**, 17.7 μM), surangin C (**13**, 5.9 μM), and mammeas A/AA (**17**, 19.5 μM), E/BB (**22**, 16.8 μM), and A/AA cyclo F (**34**, 23.6 μM), were found to inhibit testosterone 5α-reductase (Table S1).

**Table 3:** IC_50_ values of coumarin constituents from *M. siamensis* on testosterone 5α-reductase.

	**IC_**50**_ (μM)**
Kayeassamin I (**1**)	>100 (37.5)^a^
Mammeasin E (**2**)	22.6
Mammeasin F (**3**)	>100 (14.9)^a^
Mammeasin A (**4**)	19.0
Mammeasin B (**5**)	24.0
Mammeasin C (**6**)	91.9
Mammeasin D (**7**)	>100 (16.4)^a^
Kayeassamin A (**8**)	>100 (20.2)^a^
Kayeassamin E (**9**)	33.8
Kayeassamin F (**10**)	15.9
Kayeassamin G (**11**)	17.7
Surangin B (**12**)	>100 (38.5)^a^
Surangin C (**13**)	5.9
Mammea A/AA (**17**)	19.5
Mammea A/AB (**18**)	>100 (23.3)^a^
Mammea A/AC (**19**)	>100 (41.5)^a^
Mammea A/AD (**20**)	>100 (30.3)^a^
Mammea E/BB (**22**)	16.8
Mammea E/BC (**23**)	>100 (19.1)^a^
Mammea A/AA cyclo D (**24**)	>100 (38.3)^a^
Mammea A/AB cyclo D (**25**)	>100 (6.7)
Mammea A/AC cyclo D (**26**)	>100 (32.0)^a^
Mammea B/AB cyclo D (**27**)	>100 (40.7)^a^
Mammea B/AC cyclo D (**28**)	>100 (27.3)^a^
Mammea E/BC cyclo D (**29**)	>100 (31.9)^a^
Deacetylmammea E/AA cyclo D (**31**)	>100 (37.1)^a^
Deacetylmammea E/BB cyclo D (**32**)	>100 (31.9)^a^
Deacetylmammea E/BC cyclo D (**33**)	>100 (40.8)^a^
Mammea A/AA cyclo F (**34**)	23.6
Mammea A/AC cyclo F (**35**)	83.8
Finasteride^b^	0.12

*Each value represents the mean ± S.E.M. (N = 3–4)*.

a *Values in parentheses present of control of cell viability at 100 μM*.

b *Commercial finasteride was purchased from Sigma-Aldrich Co. LLC (St. Louis, USA)*.

## Conclusions

The structures of geranylated coumarin constituents, kayeassamin I (**1**) and mammeasins E (**2**) and F (**3**), newly isolated from the methanol extract of the flowers of *M. siamensis*, were determined. Of the isolated coumarins, mammeasins E (**2**, 22.6 μM), A (**4**, 19.0 μM), and B (**5**, 24.0 μM), kayeassamins E (**9**, 33.8 μM), F (**10**, 15.9 μM), and G (**11**, 17.7 μM), surangin C (**13**, 5.9 μM), and mammeas A/AA (**17**, 19.5 μM), E/BB (**22**, 16.8 μM), and A/AA cyclo F (**34**, 23.6 μM) were active 5α-reductase inhibitors. Although the intensity of the 5α-reductase inhibitory activity of these coumarins is moderate compared to a positive control having a steroid skeleton finasteride, to the best of our knowledge, there are few reports of the 5α-reductase inhibitors with non-steroidal skeletons (Dörsam and Altwein, 2009; Aggarwal et al., 2010; Chaudhary and Turner, 2010; Wu and Kapoor, 2013). Therefore, these active coumarins may be useful candidates for seed compounds of new non-steroidal 5α-reductase inhibitors. Further studies are required to elucidate the detailed structure activity relationships as well as the mode of action including the enzymatic inhibitory activity of these coumarins.

## Data Availability Statement

All datasets generated for this study are included in the article/Supplementary Material.

## Author Contributions

TM, FL, YM, HS, SS, and KN performed the experiments. TM, OM, and KN conceived and designed the experiments. SC and YP collected and identified the plant material. TM and FL wrote the paper. All authors have approved the final version of the manuscript.

### Conflict of Interest

The authors declare that the research was conducted in the absence of any commercial or financial relationships that could be construed as a potential conflict of interest.

## Supplementary Material

These data include HPLC chromatograms of mammeasins E (**2a**, **2b**) and F (**3a**, **3b**) (Figure S1), ^1^H–^1^H COSY and HMBC correlations of **2** and **3** (Figure S2), 1D and 2D NMR spectra of **1**–**3** (Figures S3–S17), and inhibitory effects of coumarin constituents (**1**–**35**) from *M. siamensis* on testosterone 5α-reductase (Table S1).
