# Supplementary material for: Geranylated Coumarins From Thai Medicinal Plant Mammea siamensis With Testosterone 5α-Reductase Inhibitory Activity
Source: Front Chem. 2020 Mar 20;8:199. doi: 10.3389/fchem.2020.00199 (PMC7099204; doi:10.3389/fchem.2020.00199)
Supplement: Supplementary file 1 [file Data_Sheet_1.PDF]

Supplementary Material

Table of Contents

Supplementary Figure 1. HPLC chromatograms of mammeasins E (**2a**, **2b**) and F (**3a**, **3b**)

Supplementary Figure 2. ^{1}H–^{1}H COSY and HMBC correlations of **2** and **3**

Supplementary Figure 3. ^{1}H NMR (800 MHz, $CDCl_3$) spectrum of kayeassamin I (**1**)

Supplementary Figure 4. ^{13}C NMR (200 MHz, $CDCl_3$) and DEPT135 spectra of kayeassamin I (**1**)

Supplementary Figure 5. DQF-COSY spectrum of kayeassamin I (**1**)

Supplementary Figure 6. HSQC spectrum of kayeassamin I (**1**)

Supplementary Figure 7. HMBC spectrum of kayeassamin I (**1**)

Supplementary Figure 8. ^{1}H NMR (700 MHz, $CDCl_3$) spectrum of mammeasin E (**2**)

Supplementary Figure 9. ^{13}C NMR (175 MHz, $CDCl_3$) and DEPT135 spectra of mammeasin E (**2**)

Supplementary Figure 10. DQF-COSY spectrum of mammeasin E (**2**)

Supplementary Figure 11. HSQC spectrum of mammeasin E (**2**)

Supplementary Figure 12. HMBC spectrum of mammeasin E (**2**)

Supplementary Figure 13. ^{1}H NMR (800 MHz, $CDCl_3$) spectrum of mammeasin F (**3**)

Supplementary Figure 14. ^{13}C NMR (200 MHz, $CDCl_3$) and DEPT135 spectra of mammeasin F (**3**)

Supplementary Figure 15. DQF-COSY spectrum of mammeasin F (**3**)

Supplementary Figure 16. HSQC spectrum of mammeasin F (**3**)

Supplementary Figure 17. HMBC spectrum of mammeasin F (**3**)

Supplementary Table 1. Inhibitory effects of coumarin constituents from *M. siamensis* on testosterone 5α-reductase

Supplementary Figure 1. HPLC chromatograms of mammeasins E (**2a**, **2b**) and F (**3a**, **3b**)

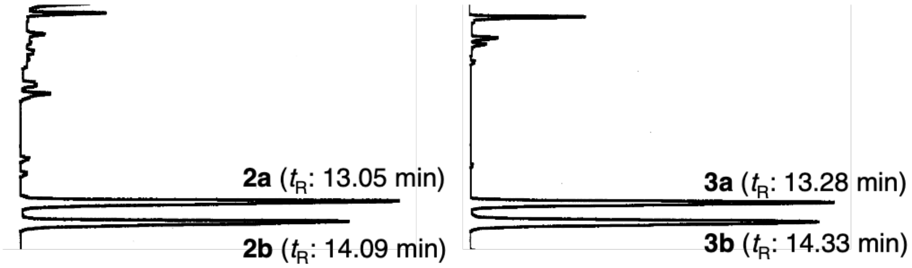

Supplementary Figure 2. ^{1}H–^{1}H COSY and HMBC correlations of **2** and **3**

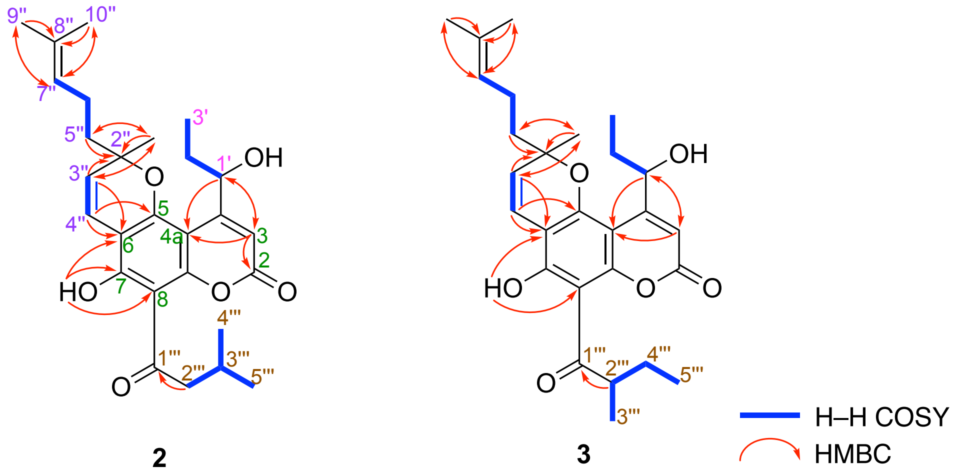

Supplementary Figure 3. ^{1}H NMR (800 MHz, $CDCl_3$) spectrum of kayeassamin I (**1**)

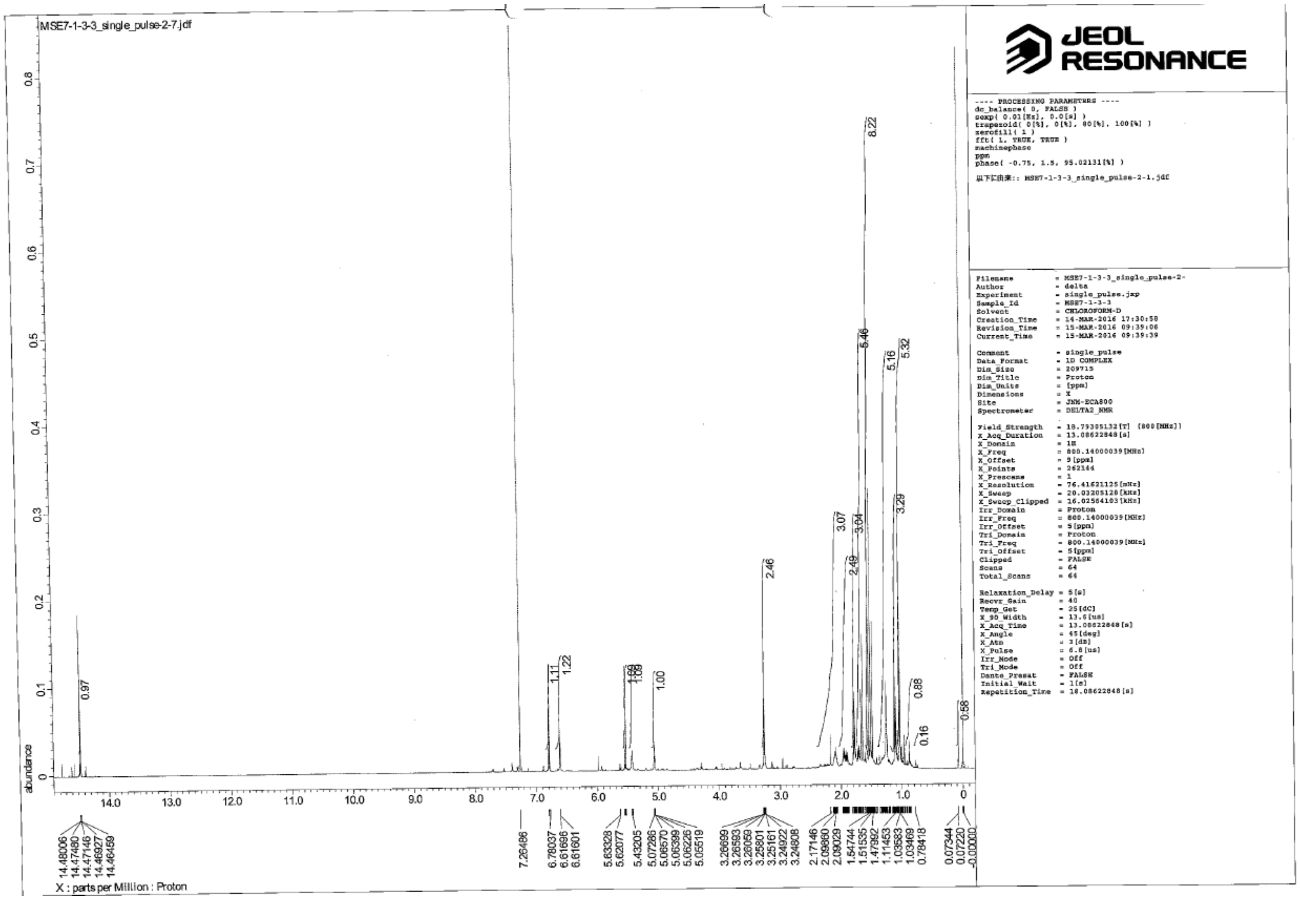

Supplementary Figure 4. ^{13}C NMR (200 MHz, $CDCl_3$) and DEPT135 spectra of kayeassamin I (**1**)

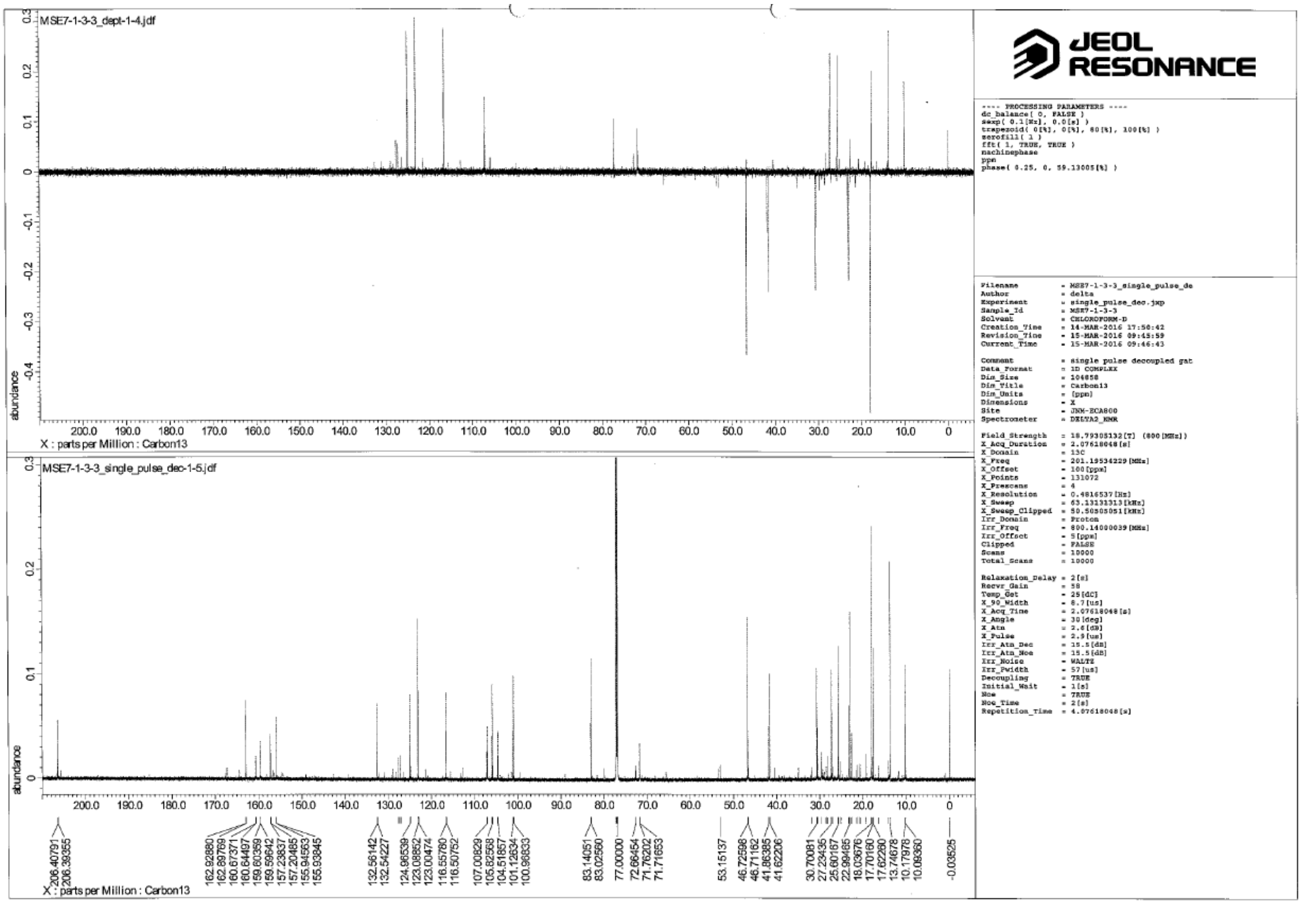

Supplementary Figure 5. DQF-COSY spectrum of kayeassamin I (**1**)

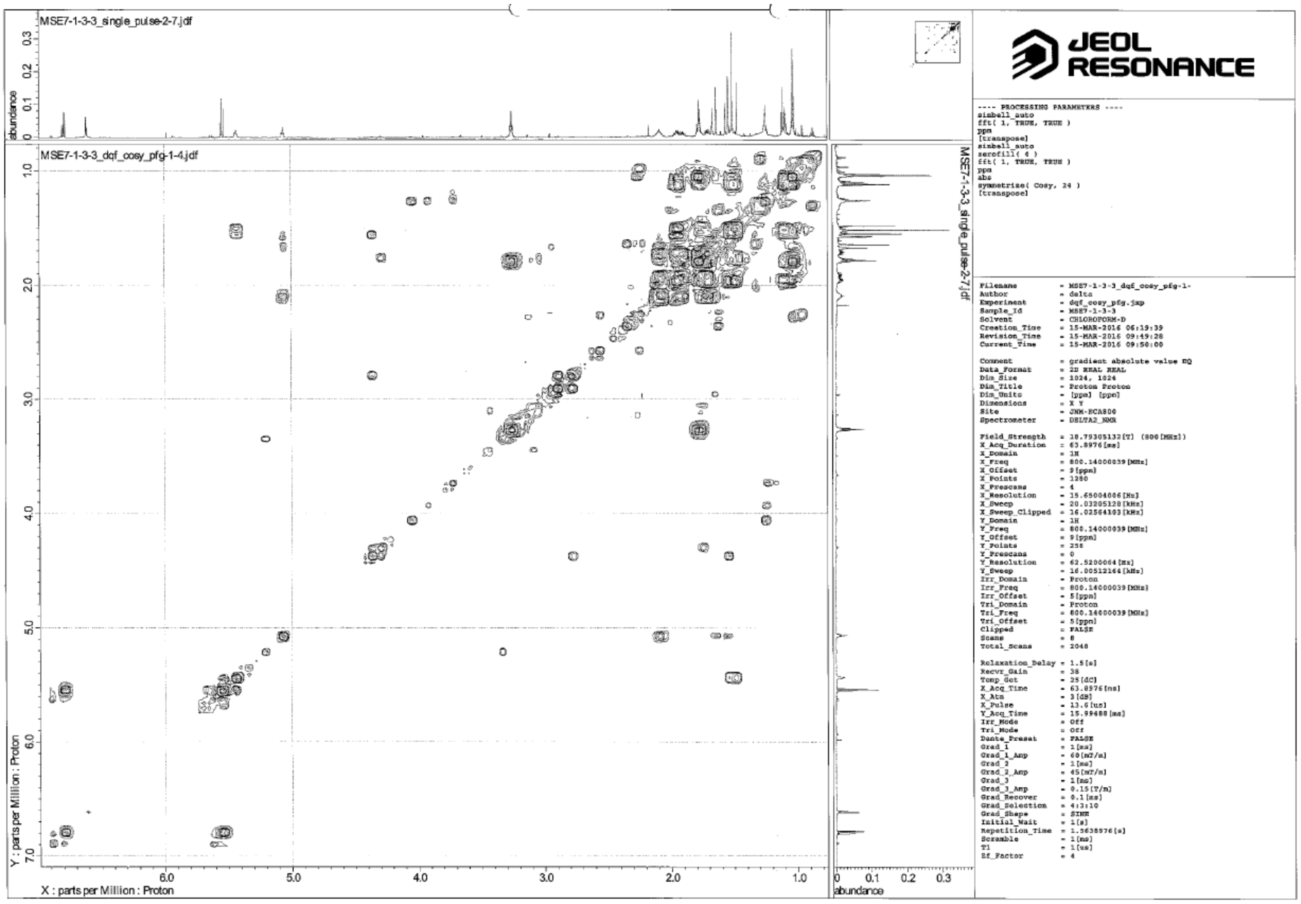

Supplementary Figure 6. HSQC spectrum of kayeassamin I (**1**)

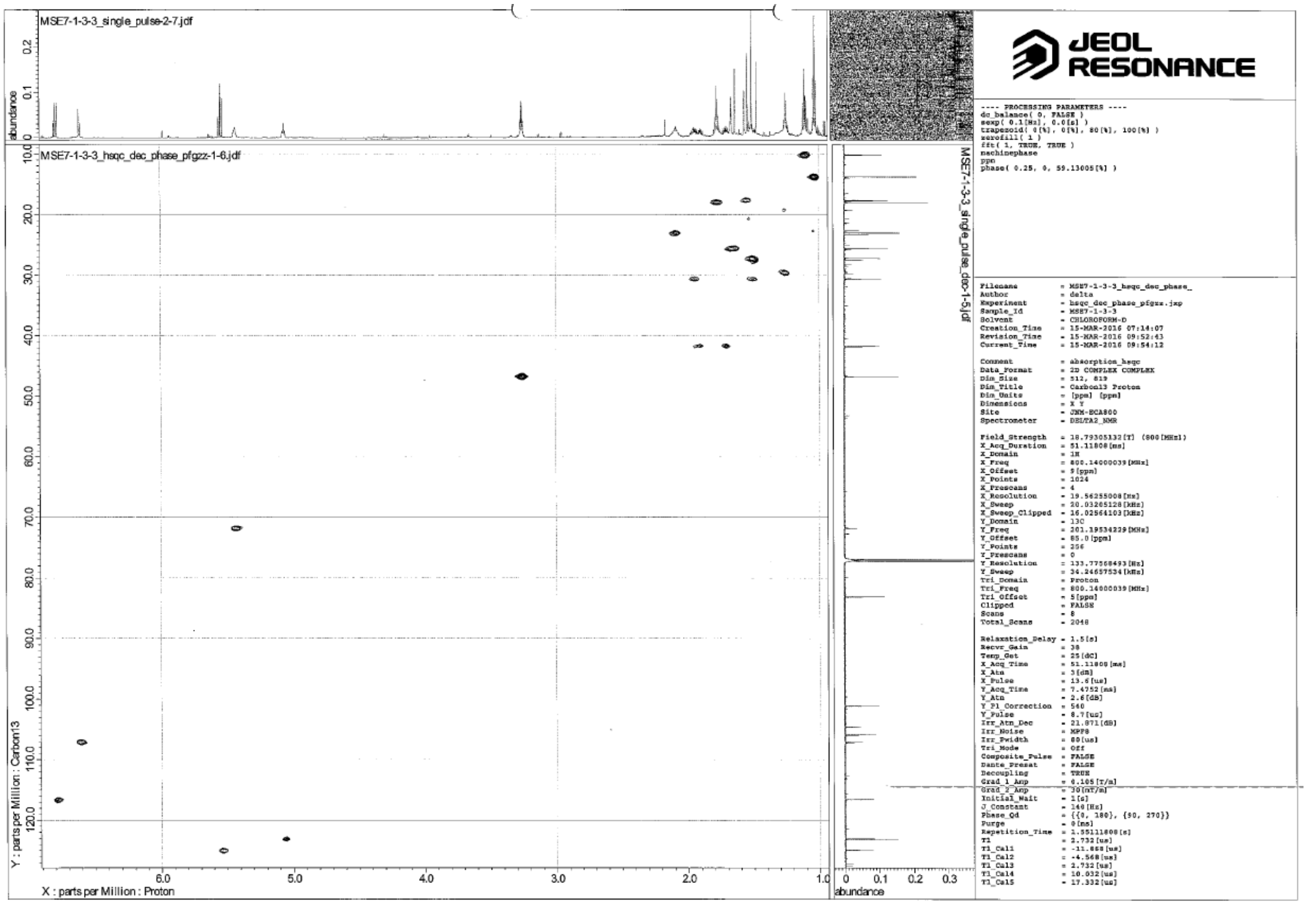

Supplementary Figure 7. HMBC spectrum of kayeassamin I (**1**)

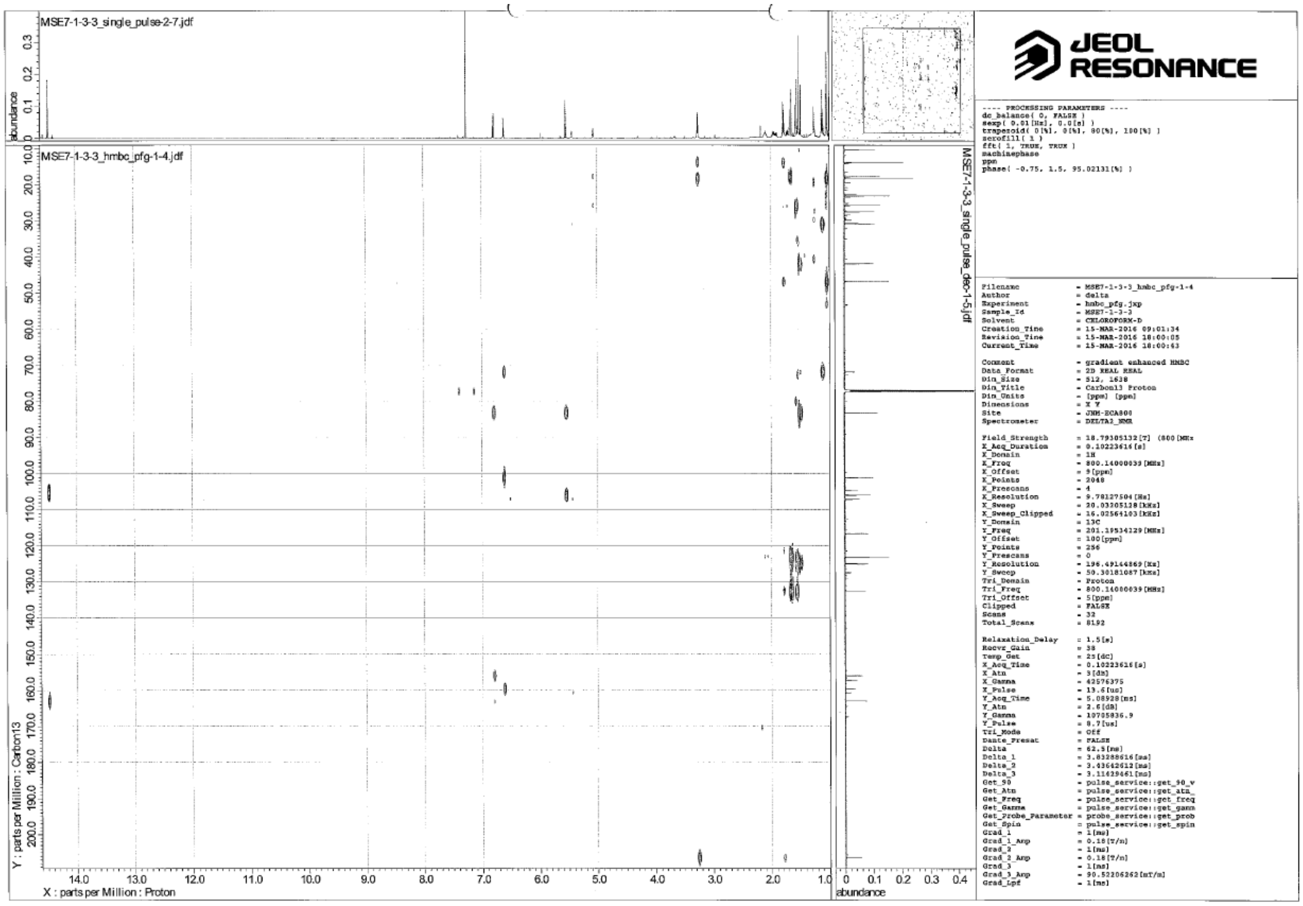

Supplementary Figure 8. ^{1}H NMR (700 MHz, $CDCl_3$) spectrum of mammeasin E (**2**)

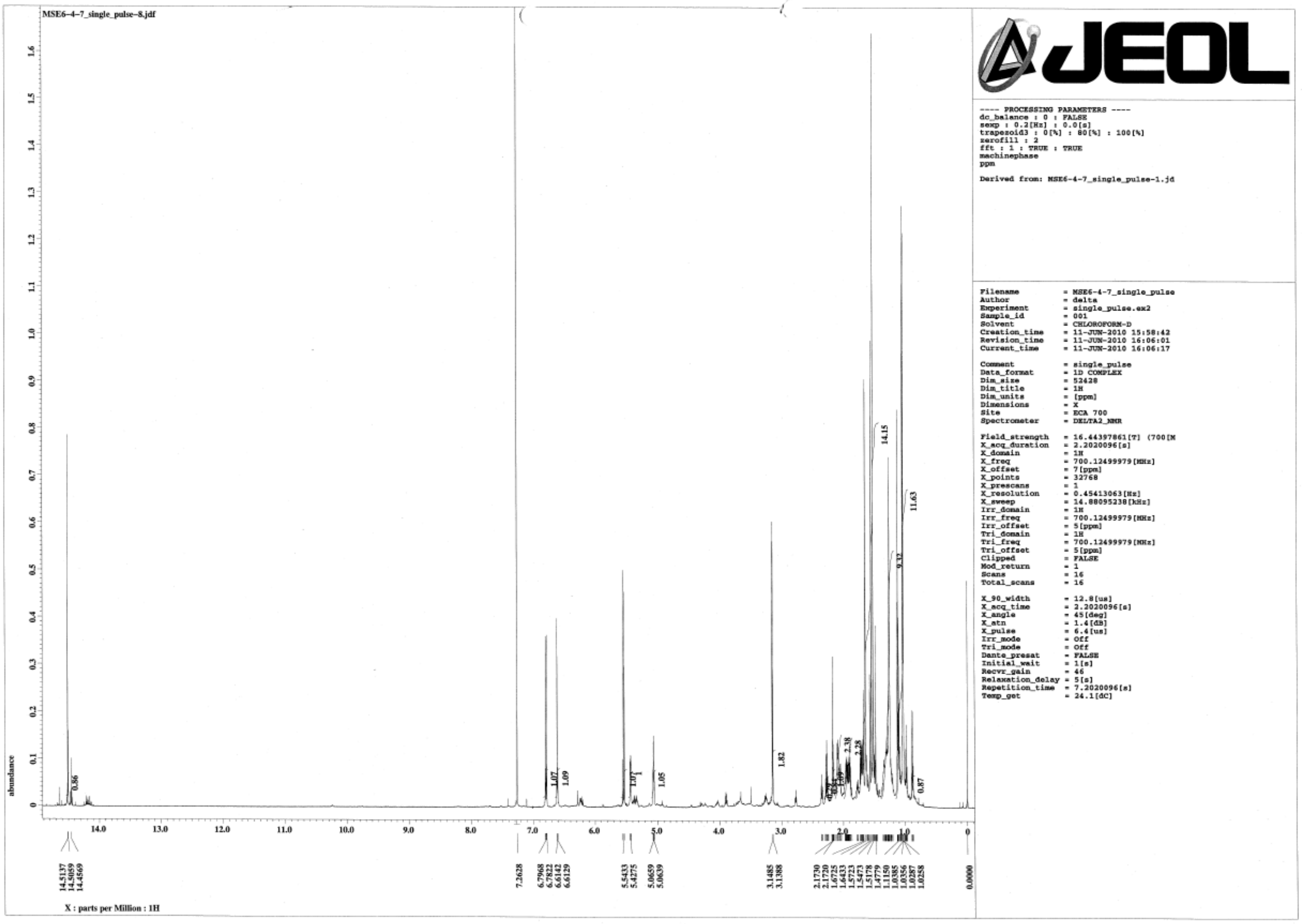

Supplementary Figure 9. ^{13}C NMR (175 MHz, $CDCl_3$) and DEPT135 spectra of mammeasin E (**2**)

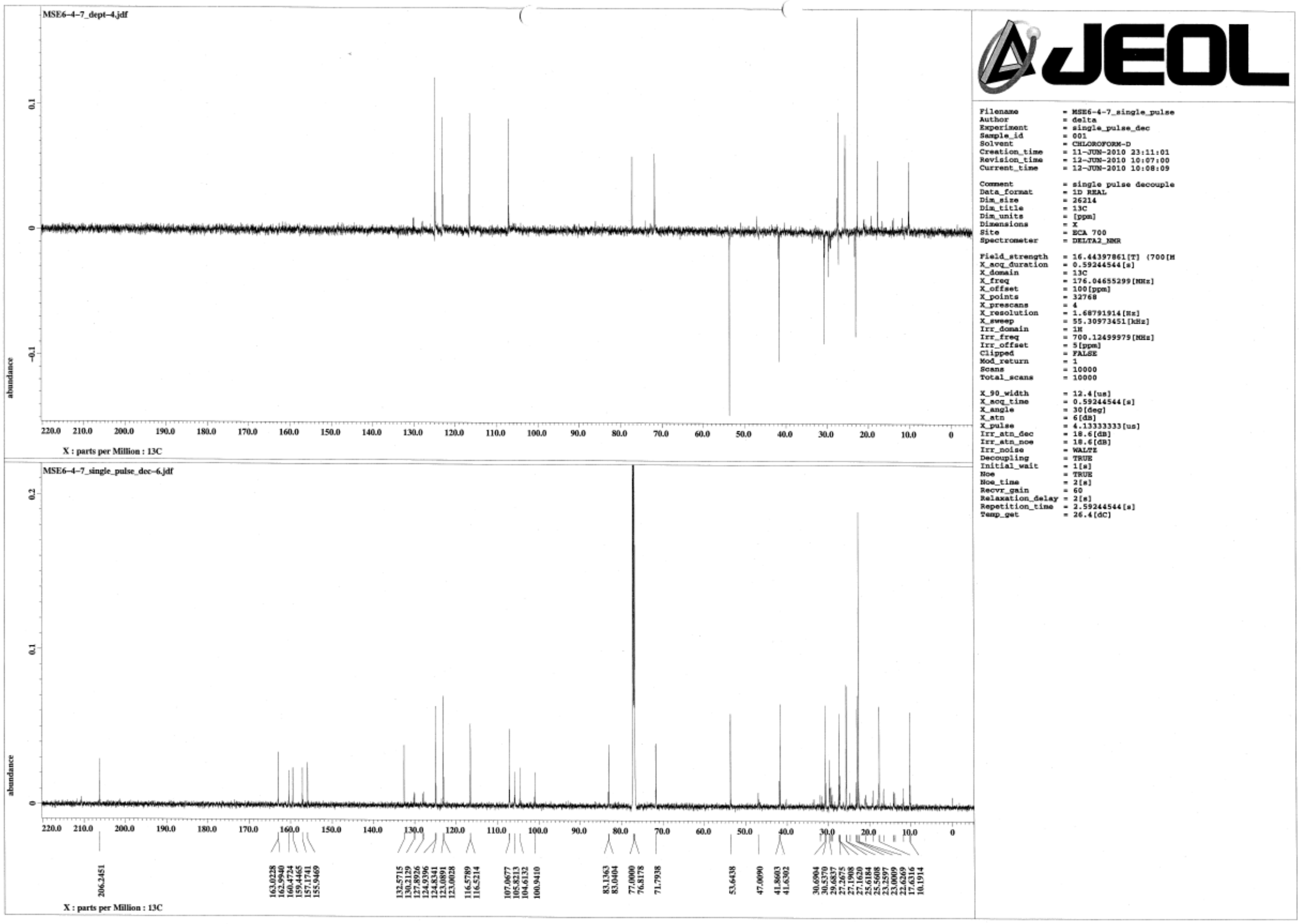

Supplementary Figure 10. DQF-COSY spectrum of mammeasin E (**2**)

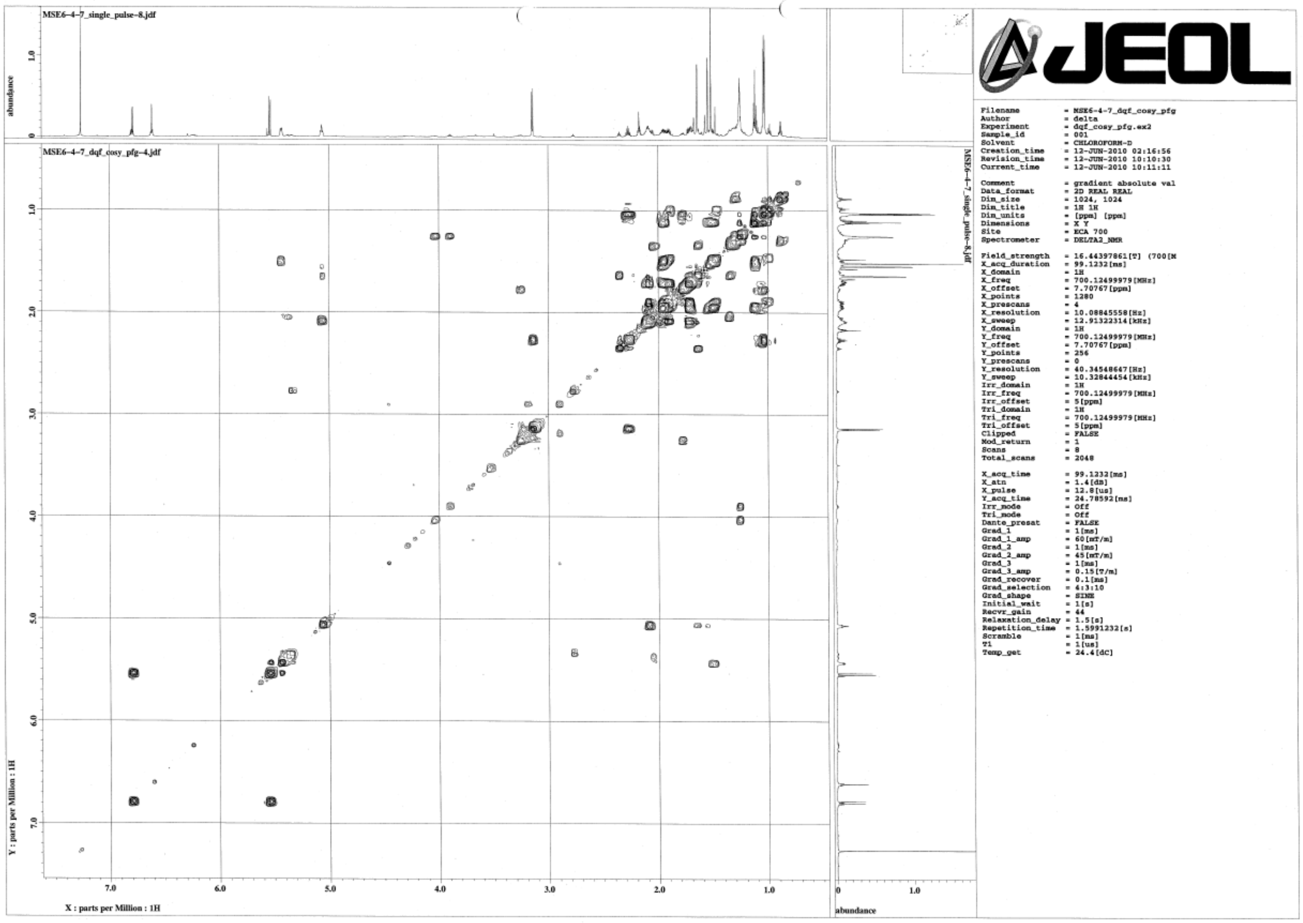

Supplementary Figure 11. HSQC spectrum of mammeasin E (**2**)

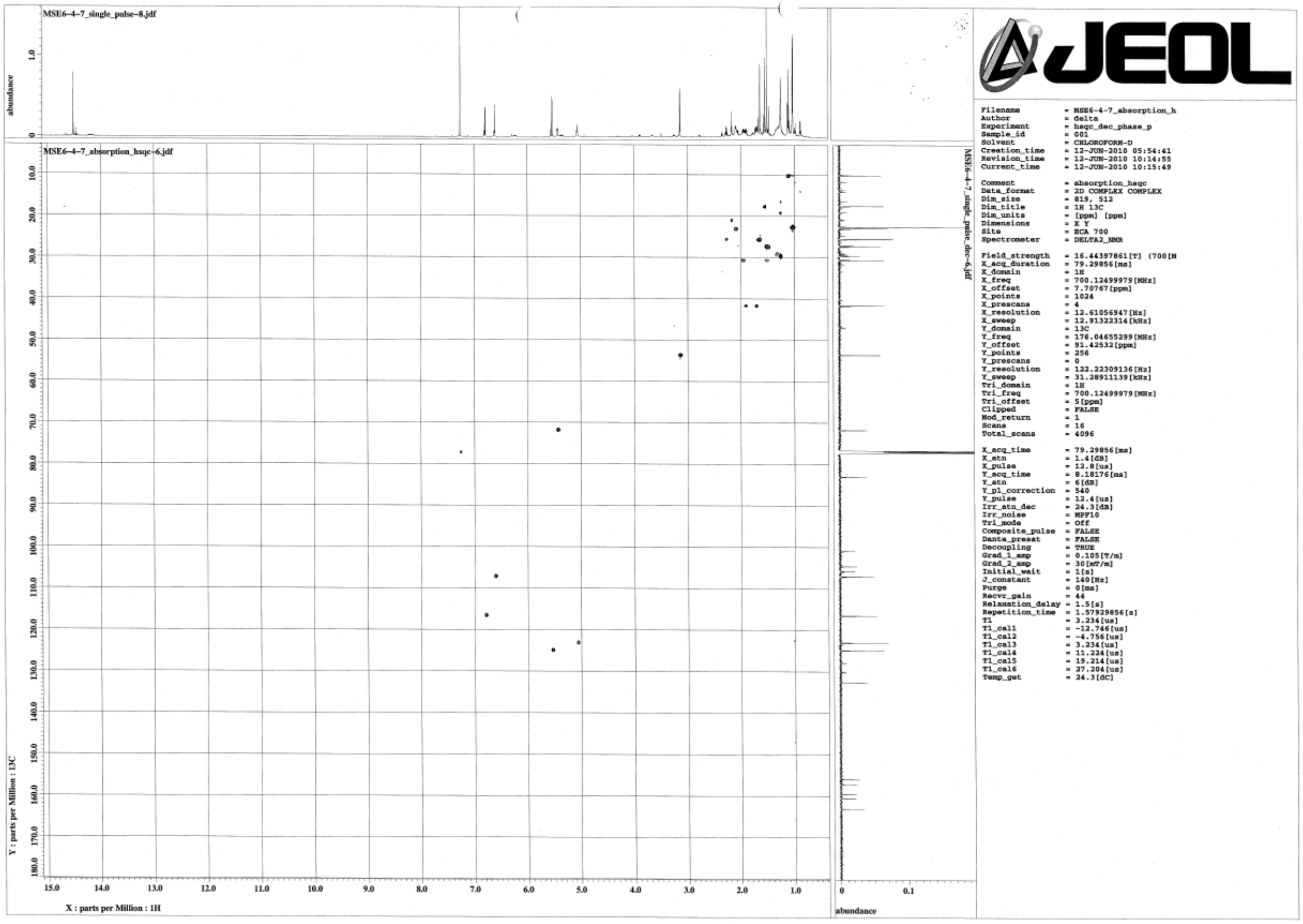

Supplementary Figure 12. HMBC spectrum of mammeasin E (**2**)

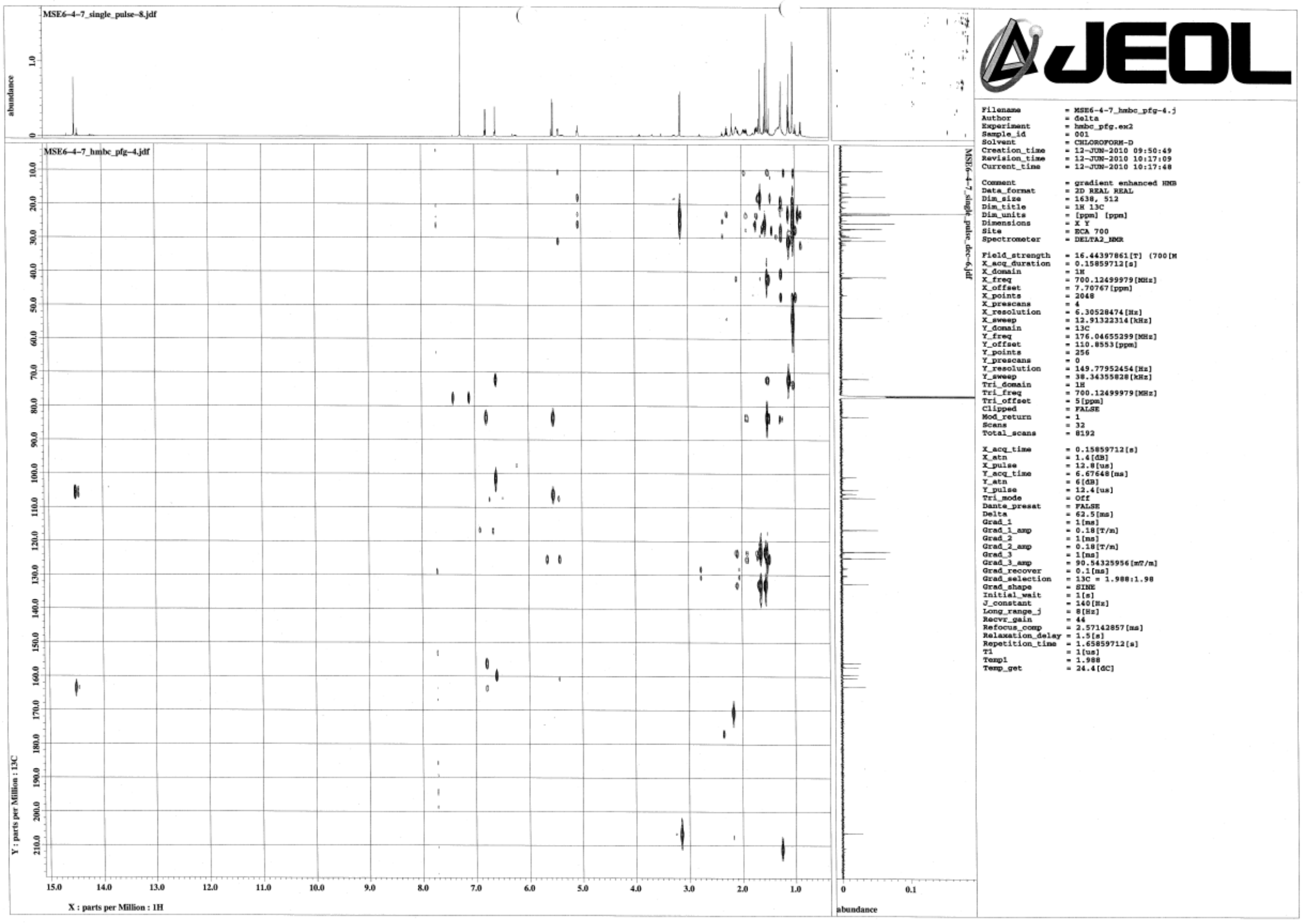

Supplementary Figure 13. ^{1}H NMR (800 MHz, $CDCl_3$) spectrum of mammeasin F (**3**)

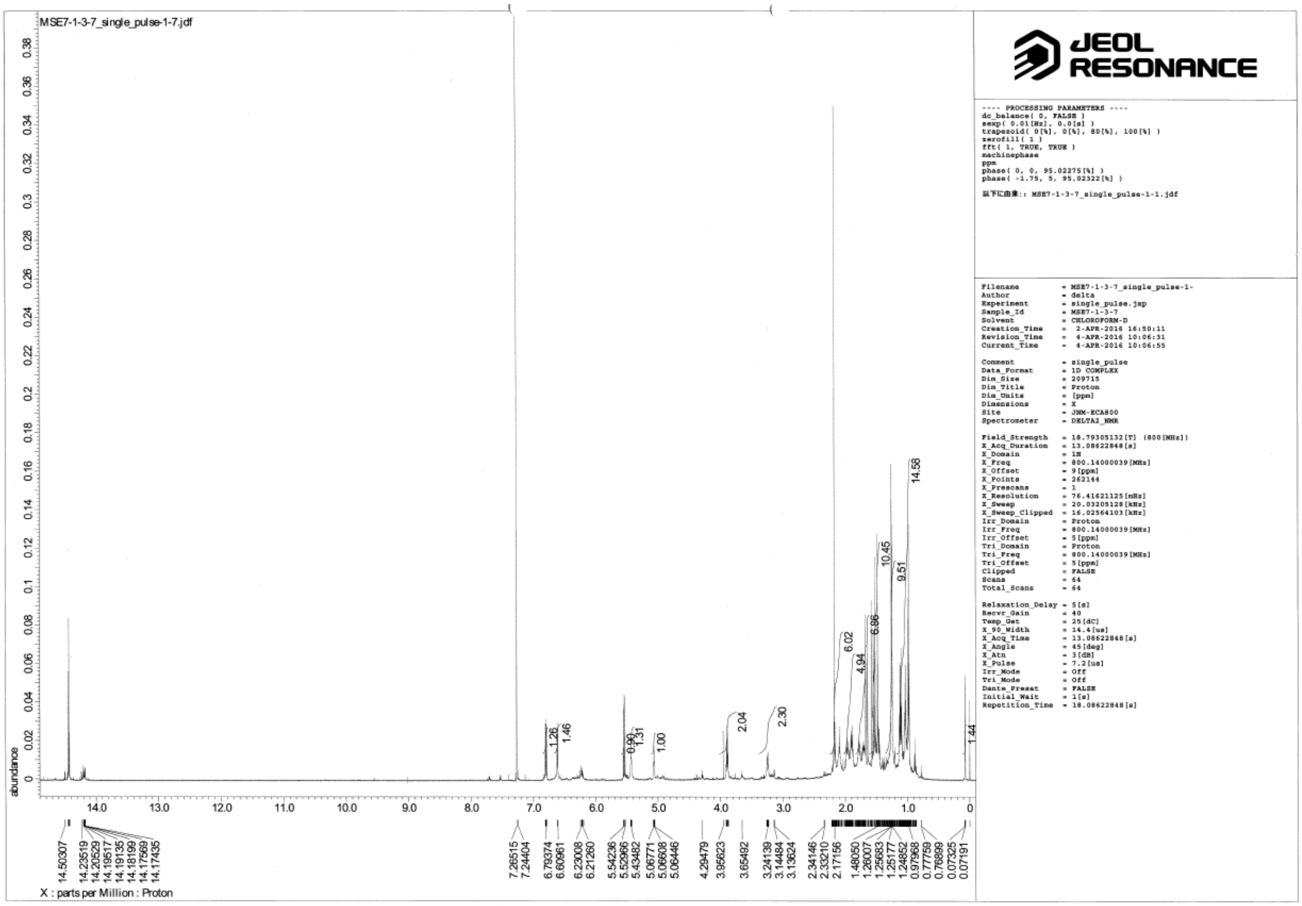

Supplementary Figure 14. ^{13}C NMR (200 MHz, $CDCl_3$) and DEPT135 spectra of mammeasin F (**3**)

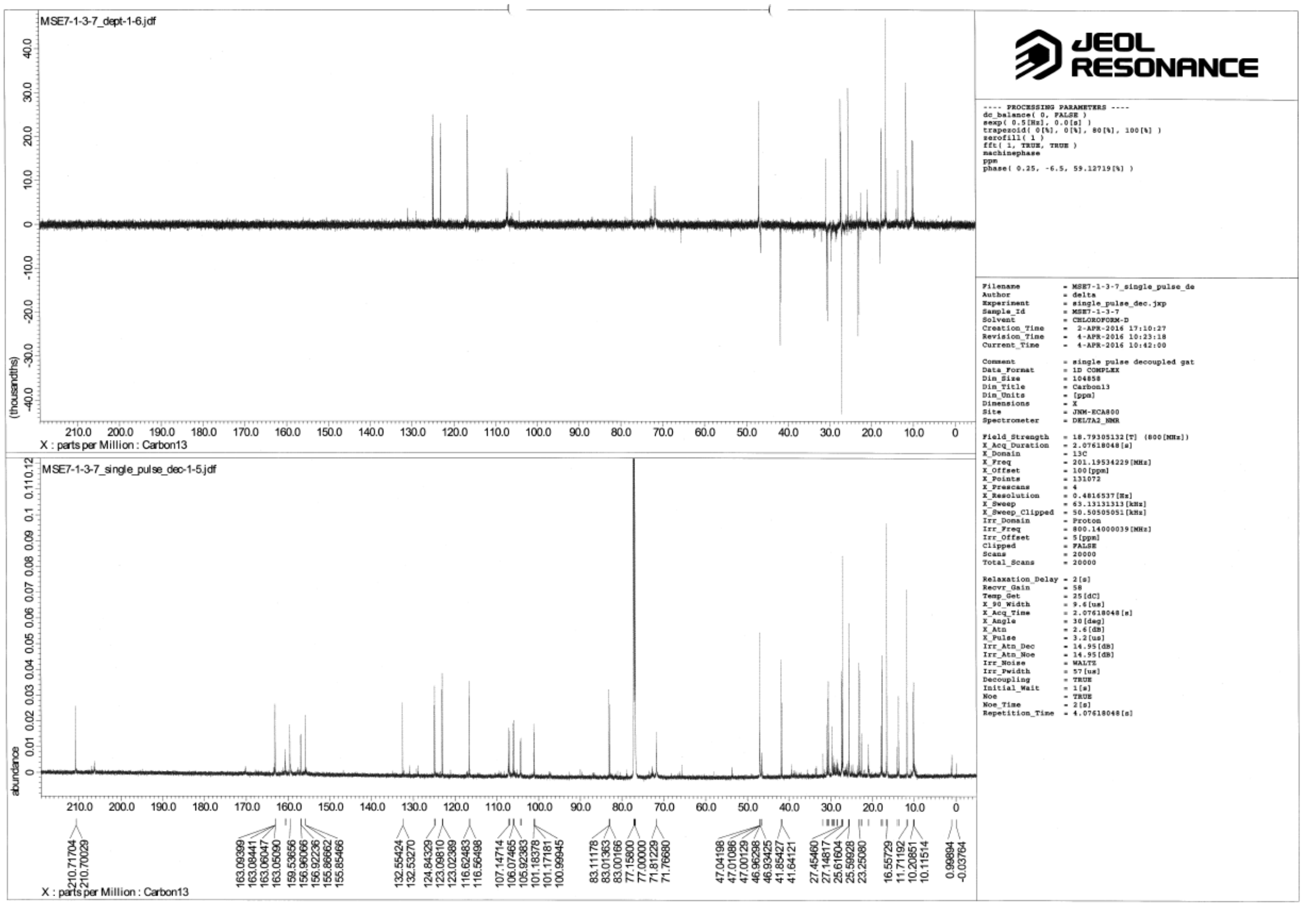

Supplementary Figure 15. DQF-COSY spectrum of mammeasin F (**3**)

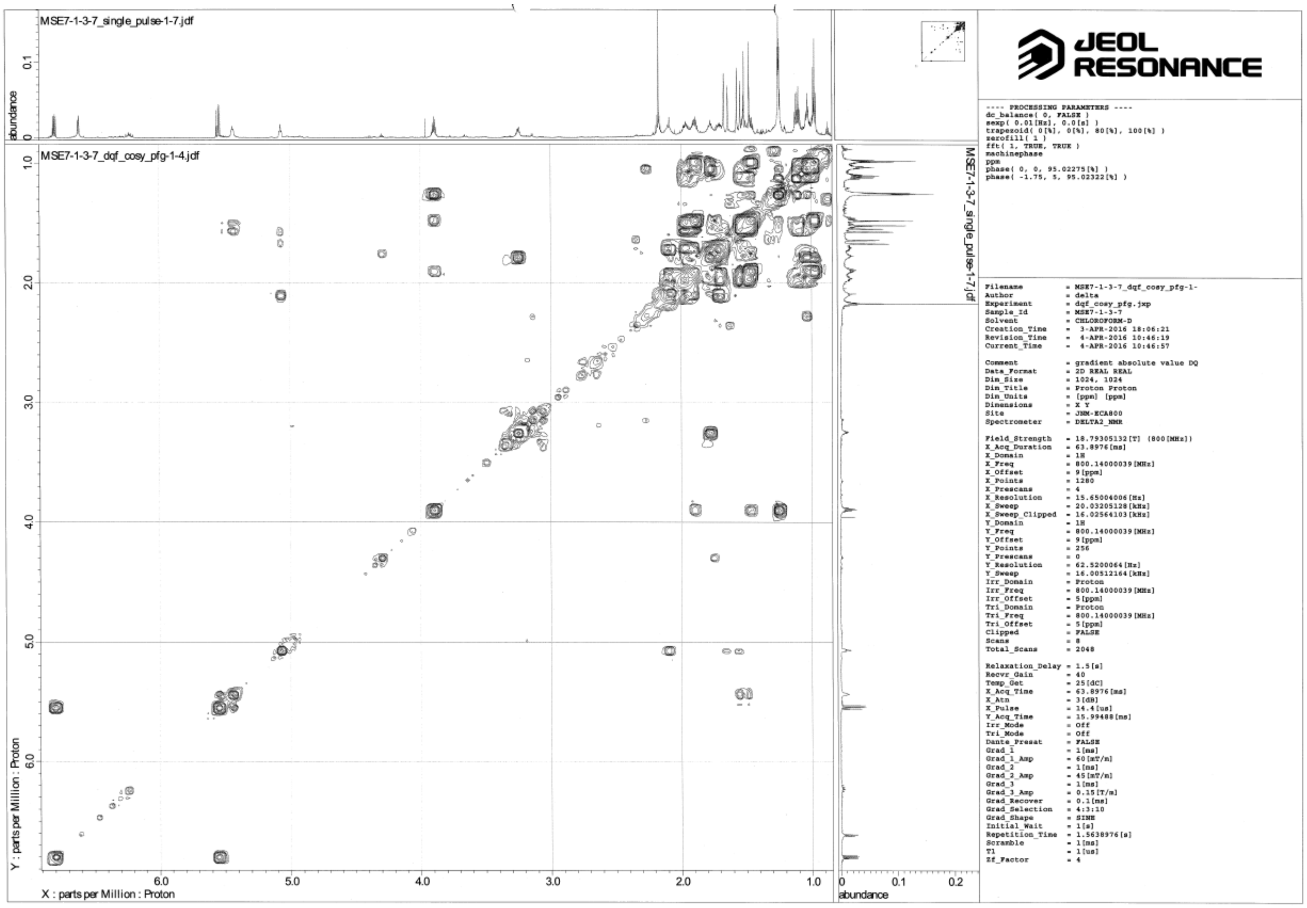

Supplementary Figure 16. HSQC spectrum of mammeasin F (**3**)

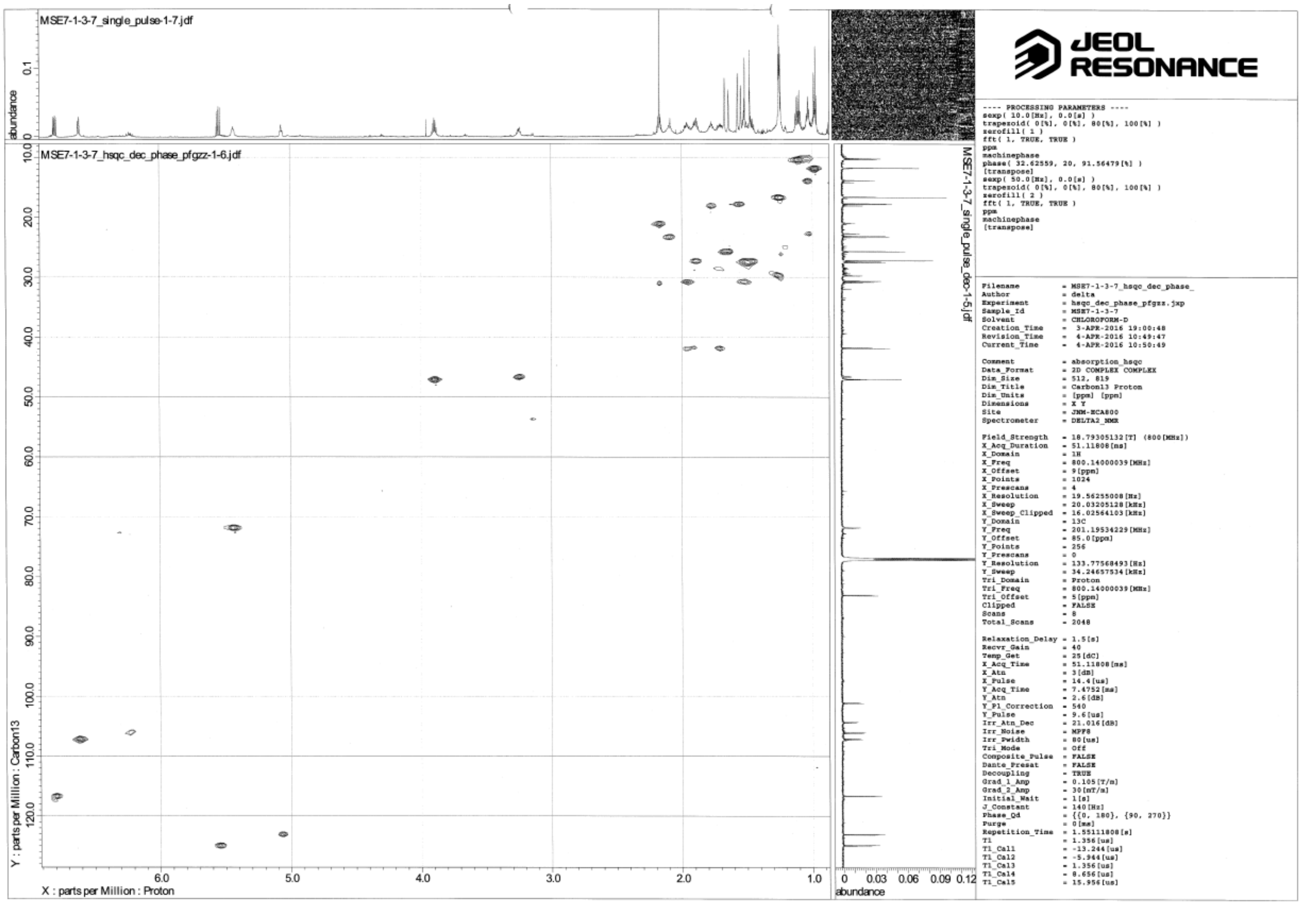

Supplementary Figure 17. HMBC spectrum of mammeasin F (**3**)

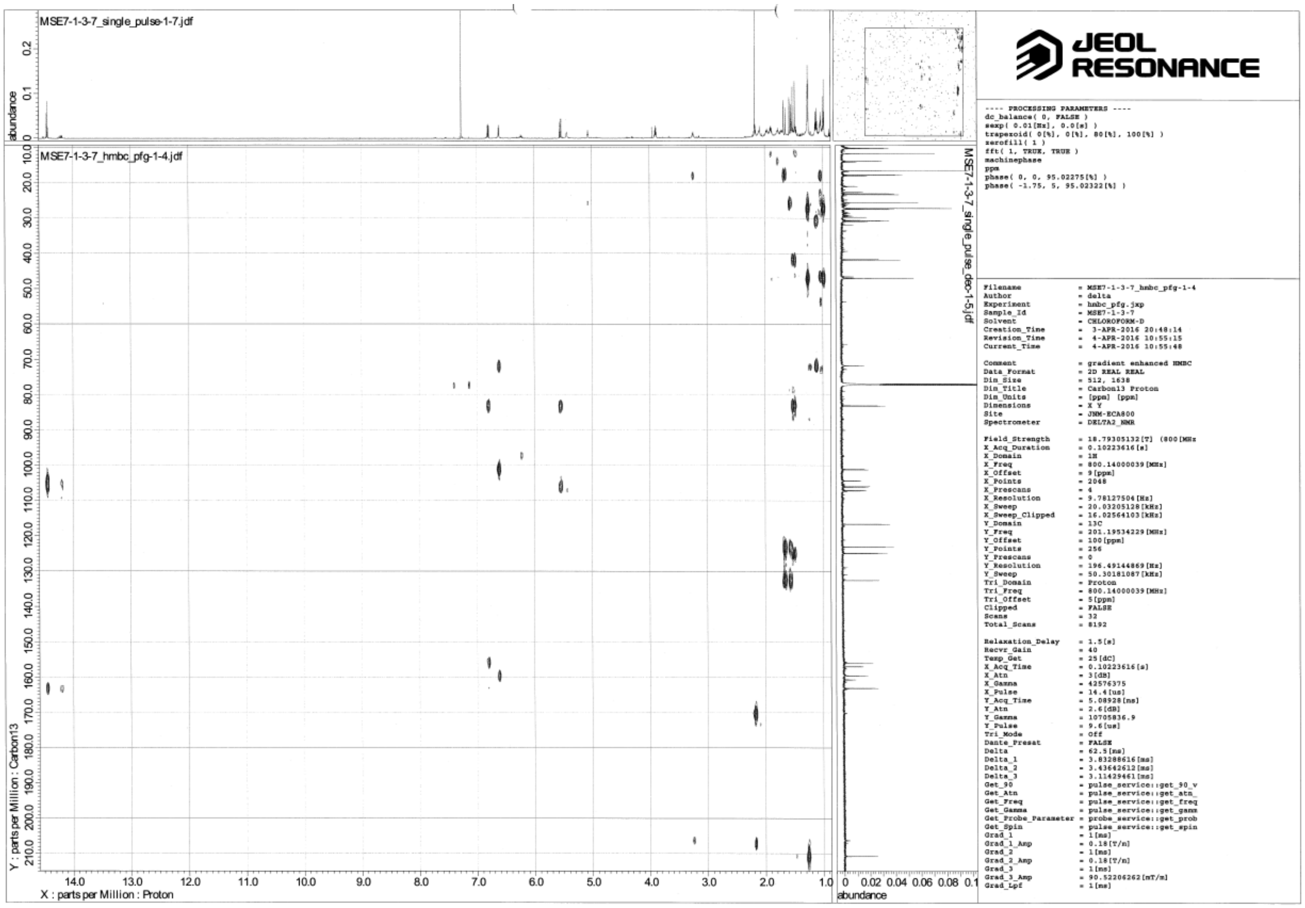

Supplementary Table 1. Inhibitory effects of coumarin constituents (**1**–**35**) from *M. siamensis* on testosterone 5α-reductase

	Inhibition (%)					IC_{50}
	0 μM	3 μM	10 μM	30 μM	100 μM	(μM)
Kayeassamin I (**1**)	0.0 ± 6.5	–	–	–	37.5 ± 11.1	>100 (37.5)[a]
Mammeasin E (**2**)	0.0 ± 6.0	20.0 ± 9.2	30.9 ± 9.3	53.9 ± 12.7	89.5 ± 15.8	22.6
Mammeasin F (**3**)	0.0 ± 5.2	–	–	–	14.9 ± 5.4	>100 (14.9)[a]
Mammeasin A (**4**)	0.0 ± 7.2	12.6 ± 10.5	40.8 ± 12.8	53.1 ± 5.2	129.0 ± 20.9	19.0
Mammeasin B (**5**)	0.0 ± 11.8	35.3 ± 6.1	39.9 ± 4.2	58.8 ± 9.6	83.7 ± 4.3	24.0
Mammeasin C (**6**)	0.0 ± 15.3	3.4 ± 13.1	8.8 ± 10.0	17.9 ± 10.6	52.9 ± 22.5	91.9
Mammeasin D (**7**)	0.0 ± 15.6	–	–	–	16.4 ± 5.8	>100 (16.4)[a]
Kayeassamin A (**8**)	0.0 ± 2.9	–	–	–	20.2 ± 7.9	>100 (20.2)[a]
Kayeassamin E (**9**)	0.0 ± 4.9	2.6 ± 7.1	11.5 ± 12.7	51.1 ± 12.4	79.9 ± 12.7	33.8
Kayeassamin F (**10**)	0.0 ± 6.0	4.6 ± 1.1	41.0 ± 6.4	68.6 ± 3.9	94.6 ± 1.3	15.9
Kayeassamin G (**11**)	0.0 ± 4.6	14.4 ± 9.7	37.5 ± 6.9	61.3 ± 3.0	62.3 ± 3.2	17.7
Surangin B (**12**)	0.0 ± 3.2	–	–	–	38.5 ± 5.0	>100 (38.5)[a]
Surangin C (**13**)	0.0 ± 1.8	28.2 ± 7.7	63.6 ± 8.3	113.6 ± 6.7	123.7 ± 9.6	5.9
Mammea A/AA (**17**)	0.0 ± 4.0	18.9 ± 7.0	32.0 ± 3.9	62.0 ± 2.8	92.9 ± 5.3	19.5
Mammea A/AB (**18**)	0.0 ± 3.2	–	–	–	23.3 ± 7.2	>100 (23.3)[a]
Mammea A/AC (**19**)	0.0 ± 3.2	–	–	–	41.5 ± 11.4	>100 (41.5)[a]
Mammea A/AD (**20**)	0.0 ± 3.2	–	–	–	30.3 ± 2.1	>100 (30.3)[a]
Mammea E/BB (**22**)	0.0 ± 6.0	8.4 ± 6.1	43.3 ± 3.8	58.0 ± 4.5	93.4 ± 4.8	16.8
Mammea E/BC (**23**)	0.0 ± 3.2	–	–	–	19.1 ± 1.3	>100 (19.1)[a]
Mammea A/AA cyclo D (**24**)	0.0 ± 2.9	–	–	–	38.3 ± 3.8	>100 (38.3)[a]
Mammea A/AB cyclo D (**25**)	0.0 ± 6.0	–	–	–	6.7 ± 4.5	>100 (6.7)[a]
Mammea A/AC cyclo D (**26**)	0.0 ± 1.7	–	–	–	32.0 ± 1.9	>100 (32.0)[a]
Mammea B/AB cyclo D (**27**)	0.0 ± 4.6	–	–	–	40.7 ± 18.1	>100 (40.7)[a]
Mammea B/AC cyclo D (**28**)	0.0 ± 1.7	–	–	–	27.3 ± 2.0	>100 (27.3)[a]
Mammea E/BC cyclo D (**29**)	0.0 ± 2.9	–	–	–	31.9 ± 3.9	>100 (31.9)[a]
Deacetylmammea E/AA cyclo D (**31**)	0.0 ± 2.9	–	–	–	37.1 ± 4.0	>100 (37.1)[a]
Deacetylmammea E/BB cyclo D (**32**)	0.0 ± 2.9	–	–	–	31.9 ± 8.8	>100 (31.9)[a]
Deacetylmammea E/BC cyclo D (**33**)	0.0 ± 1.7	–	–	–	40.8 ± 4.1	>100 (40.8)[a]
Mammea A/AA cyclo F (**34**)	0.0 ± 8.6	13.8 ± 6.7	21.6 ± 10.3	62.8 ± 18.1	57.0 ± 15.7	23.6
Mammea A/AC cyclo F (**35**)	0.0 ± 5.3	4.2 ± 6.1	18.4 ± 6.9	32.5 ± 6.0	54.8 ± 23.2	83.8

	Inhibition (%)					IC_{50}
	0 μM	0.1 μM	0.3 μM	1 μM	3 μM	(μM)
Finasteride[b]	0.0 ± 6.4	48.5 ± 10.9	61.8 ± 11.4	76.0 ± 9.8	91.3 ± 13.1	0.12

Each value represents the mean ± S.E.M. (N = 3–4).
[a] Values in parentheses present of control of cell viability at 100 μM.
[b] Commercial finasteride was purchased from Sigma-Aldrich Co. LLC (St. Louis, USA).
